# Supplementary material for: A structure-based mRNA vaccine for Nipah virus in healthy adults: a phase 1 trial
Source: Nat Med. 2026 Mar 12;32(4):1401–10. doi: 10.1038/s41591-026-04265-1 (PMC13099371; doi:10.1038/s41591-026-04265-1)
Supplement: Supplementary file 1 — Supplementary Tables 1–7 and Protocol. [file 41591_2026_4265_MOESM1_ESM.pdf]

nature medicine

Supplementary information https://doi.org/10.1038/s41591-026-04265-1

A structure-based mRNA vaccine for Nipah virus in healthy adults: a phase 1 trial

In the format provided by the authors and unedited

Supplementary Appendix

Table of Contents

**Table of Contents**	**Page**
Table S1	2
Table S2	3
Table S3	4
Table S4	5
Table S5	6
Table S6	7
Table S7	8
Study Protocol	9

Table S1: P-values of Pairwise Comparisons of Binding IgG Antibody Against NiV(M) Pre-F Protein ELISA GMTs Between Study Groups by Time Point

Time Point	Study Group	25 mcg mRNA-1215	50 mcg mRNA-1215	100 mcg mRNA-1215
		P-value		
Week 24	10 mcg mRNA-1215	0.023	0.008	0.032
	25 mcg mRNA-1215	-	0.656	0.892
	50 mcg mRNA-1215	-	-	0.562
Week 56	10 mcg mRNA-1215	0.006	0.001	0.019
	25 mcg mRNA-1215	-	0.423	0.636
	50 mcg mRNA-1215	-	-	0.211

p-values were determined by uncorrected pairwise two-sided t-test; adjustments for multiple comparisons were not performed.

Table S2: P-values of Pairwise Comparisons of Binding IgG Antibody Against NiV(M) G Protein ELISA GMTs Between Study Groups by Time Point

Time Point	Study Group	25 mcg mRNA-1215	50 mcg mRNA-1215	100 mcg mRNA-1215
		P-value		
**Week 24**	10 mcg mRNA-1215	0.007	0.001	0.001
	25 mcg mRNA-1215	-	0.536	0.416
	50 mcg mRNA-1215	-	-	0.844
**Week 56**	10 mcg mRNA-1215	0.007	0.001	0.001
	25 mcg mRNA-1215	-	0.306	0.479
	50 mcg mRNA-1215	-	-	0.734

p-values were determined by uncorrected pairwise two-sided t-test; adjustments for multiple comparisons were not performed.

Table S3: P-values of Pairwise Comparisons of NiV(M) Pseudovirus Neutralization Assay GMTs Between Study Groups by Time Point

Time Point	Study Group	25 mcg mRNA-1215	50 mcg mRNA-1215	100 mcg mRNA-1215
		P-value		
**Week 24**	10 mcg mRNA-1215	0.014	0.004	0.002
	25 mcg mRNA-1215	-	0.592	0.443
	50 mcg mRNA-1215	-	-	0.816
**Week 56**	10 mcg mRNA-1215	0.060	0.006	0.032
	25 mcg mRNA-1215	-	0.283	0.771
	50 mcg mRNA-1215	-	-	0.427

p-values were determined by uncorrected pairwise two-sided t-test; adjustments for multiple comparisons were not performed.

Table S4: P-values of Pairwise Comparisons of NiV(B) Pseudovirus Neutralization Assay GMTs Between Study Groups by Time Point

Time Point	Study Group	25 mcg mRNA-1215	50 mcg mRNA-1215	100 mcg mRNA-1215
		P-value		
Week 24	10 mcg mRNA-1215	0.006	0.003	0.005
	25 mcg mRNA-1215	-	0.802	0.961
	50 mcg mRNA-1215	-	-	0.840

p-values were determined by uncorrected pairwise two-sided t-test; adjustments for multiple comparisons were not performed.

Table S5: P-values of Pairwise Comparisons of Binding IgG Antibody Against HeV Pre-F Protein ELISA GMTs Between Study Groups by Time Point

Time Point	Study Group	25 mcg mRNA-1215	50 mcg mRNA-1215	100 mcg mRNA-1215
		P-value		
Week 12	10 mcg mRNA-1215	0.021	0.003	0.009
	25 mcg mRNA-1215	-	0.432	0.740
	50 mcg mRNA-1215	-	-	0.648
Week 24	10 mcg mRNA-1215	0.008	0.002	0.005
	25 mcg mRNA-1215	-	0.572	0.874
	50 mcg mRNA-1215	-	-	0.684
Week 56	10 mcg mRNA-1215	0.003	0.002	0.011
	25 mcg mRNA-1215	-	0.803	0.635
	50 mcg mRNA-1215	-	-	0.478

p-values were determined by uncorrected pairwise two-sided t-test; adjustments for multiple comparisons were not performed.

Table S6: P-values of Pairwise Comparisons of Binding IgG Antibody Against HeV G Protein ELISA GMTs Between Study Groups by Time Point

Time Point	Study Group	25 mcg mRNA-1215	50 mcg mRNA-1215	100 mcg mRNA-1215
		P-value		
**Week 12**	10 mcg mRNA-1215	0.008	0.002	< 0.001
	25 mcg mRNA-1215	-	0.530	0.275
	50 mcg mRNA-1215	-	-	0.639
**Week 24**	10 mcg mRNA-1215	0.005	0.001	0.001
	25 mcg mRNA-1215	-	0.667	0.494
	50 mcg mRNA-1215	-	-	0.797
**Week 56**	10 mcg mRNA-1215	0.025	0.014	0.012
	25 mcg mRNA-1215	-	0.751	0.749
	50 mcg mRNA-1215	-	-	0.995

p-values were determined by uncorrected pairwise two-sided t-test; adjustments for multiple comparisons were not performed.

Table S7: P-values of Pairwise Comparisons of HeV Pseudovirus Neutralization Assay GMTs Between Study Groups by Time Point

Time Point	Study Group	25 mcg mRNA-1215	50 mcg mRNA-1215	100 mcg mRNA-1215
		P-value		
Week 12	10 mcg mRNA-1215	0.002	0.001	<0.001
	25 mcg mRNA-1215	-	0.824	0.570
	50 mcg mRNA-1215	-	-	0.729
Week 24	10 mcg mRNA-1215	0.009	0.005	0.006
	25 mcg mRNA-1215	-	0.792	0.885
	50 mcg mRNA-1215	-	-	0.905

p-values were determined by uncorrected pairwise two-sided t-test; adjustments for multiple comparisons were not performed.

Protocol Title: VRC 322 (000687): A Phase I, Dose Escalation, Open-Label Clinical Trial to Evaluate Safety, Tolerability and Immunogenicity of a Nipah Virus (NiV) mRNA Vaccine, mRNA-1215, in Healthy Adults

NCT:05398796

Documents:

- IRB-approved Protocol (v5.0 20DEC2023) - Statistical Analysis Considerations located in Section 6 of the Protocol
- Final IRB-approved Main Informed Consent (v4.0 20DEC2023) -
  IRB Approval/Document Date:28DEC2023

VACCINE RESEARCH CENTER

A Phase I, Dose Escalation, Open-Label Clinical Trial to Evaluate Safety, Tolerability and Immunogenicity of a Nipah Virus (NiV) mRNA Vaccine, mRNA-1215, in Healthy Adults

Abbreviated Title: NiV mRNA-1215 vaccine

DMID Protocol Number: 21-0016

NIH 000687

IND 28335

IND Sponsor: Division of Microbiology and Infectious Diseases (DMID)

Investigational Product, mRNA-1215, Manufacturer: ModernaTX

Version Number: 5.0

December 20, 2023

NIH Principal Investigator: Lesia Dropulic, M.D.
VRC/NIAID
[redacted]

STATEMENT OF COMPLIANCE

National Institutes of Health (NIH)-funded investigators and clinical trial site staff who are responsible for the conduct, management, or oversight of NIH-funded clinical trials have completed Human Subjects Protection and ICH GCP Training.

The protocol, informed consent form(s), recruitment materials, and all participant materials will be submitted to the Institutional Review Board (IRB) for review and approval. Approval of both the protocol and the consent form must be obtained before any participant is enrolled. Any amendment to the protocol will require review and approval by the IRB before the changes are implemented to the study. In addition, all changes to the consent form will be IRB-approved; a determination will be made regarding whether a new consent needs to be obtained from participants who provided consent, using a previously approved consent form.

The study will be carried out in accordance with the following as applicable:

- United States Code of Federal Regulations (CFR) 45 CFR Part 46: Protection of Human Subjects
- Food and Drug Administration (FDA) Regulations: 21 CFR Part 50 (Protection of Human Subjects), 21 CFR Part 54 (Financial Disclosure by Clinical Investigators), 21 CFR Part 56 (Institutional Review Boards), 21 CFR Part 11, and 21 CFR Part 312 (Investigational New Drug Application), and/or 21 CFR 812 (Investigational Device Exemptions)
- The International Council for Harmonisation of Technical Requirements for Registration of Pharmaceuticals for Human Use (ICH) E6(R2) Good Clinical Practice, and the Belmont Report: Ethical Principles and Guidelines for the Protection of Human Subjects of Research, Report of the National Commission for the Protection of Human Subjects of Biomedical and Behavioral Research
- The policies and procedures of National Institutes of Health (NIH) Office of Extramural Research and DMID
- The National Institute of Allergy and Infectious Diseases (NIAID)
- Any additional Federal, State, and Local Regulations and Guidance

PROTOCOL SIGNATURE PAGE

VRC 322/DMID 21-0016: A Phase I, Dose Escalation, Open-Label Clinical Trial to Evaluate Safety, Tolerability and Immunogenicity of a Nipah Virus (NiV) mRNA Vaccine, mRNA-1215, in Healthy Adults

I, the Principal Investigator for the study site indicated below, agree to conduct the study in full accordance with the provisions of this protocol and all applicable protocol-related documents. I agree to conduct the study in compliance with United States (US) Health and Human Services (HHS) regulations (45CFR 46); applicable US Food and Drug Administration (FDA) regulations; standards of the International Council for Harmonization Guidelines for Good Clinical Practice (E6); Institutional Review Board/Ethics Committee (IRB/EC) determinations; all applicable in-country, state, and local laws and regulations; and other applicable requirements (e.g., US National Institutes of Health) and institutional policies. I will comply with all requirements regarding the obligations of investigators as outlined in the Statement of Investigator (Form FDA 1572), which I have also signed. I agree to maintain all study documentation pertaining to the conduct of this study, including, but not limited to, case report forms, source documents, laboratory test results, and medication inventory records, per FDA regulation (21 CFR 312.62) and all applicable requirements. The protocol signature page will be signed for subsequent protocol approvals. No study records will be destroyed without prior authorization from VRC/NIAID.

Publication of the results of this study will be governed by the VRC/NIAID and DMID/NIAID policies. Any presentation, abstract, or manuscript will be made available by the investigators to VRC, DMID and Moderna leaderships for review prior to submission. I have read and understand the information in this protocol and will ensure that all associates, colleagues, and employees assisting in the conduct of the study are informed about the obligations incurred by their contribution to the study.

Lesia Dropulic, MD	VRC / Vaccine Evaluation Clinic (VEC)
Name/Title of Principal Investigator	Investigator Study Site Name
______________________________	______________________________
Signature of Principal Investigator	Date

TABLE OF CONTENTS

1. INTRODUCTION 12
1.1 Study Rationale 12
1.1.1 Rationale for Development of NiV mRNA-1215 Vaccine 12
1.1.2 Rationale for mRNA-1215 Vaccine Dose and Regimen 13
1.2 Background 14
1.2.1 Previous Human Experience with mRNA-based Vaccines 14
1.2.2 Preclinical Studies with mRNA-1215 Vaccine 14
1.2.3 Human Experience with mRNA-1215 Vaccine 14
1.3 Laboratory Assessment of mRNA-1215 Vaccine Immunogenicity 16
1.3.1 Interim Evaluation of the mRNA-1215 Vaccine Immunogenicity 17
2. STUDY PRODUCTS 18
2.1 mRNA-1215 Vaccine 18
2.2 Diluent: 0.9% NaCl for injection, USP 18
3. STUDY OBJECTIVES 19
3.1 Primary Objectives 19
3.2 Secondary Objectives 19
3.3 Exploratory Objectives 19
4. SUBJECT POPULATION AND CLINICAL PROCEDURES 20
4.1 Overall Study Design 20
4.2 Study Population 20
4.2.1 Inclusion Criteria 20
4.2.2 Exclusion Criteria 21
4.3 Inclusion of Vulnerable Subjects 22
4.3.1 Pregnant Women 22
4.3.2 Children 23
4.3.3 Adult Subjects who Lack the Capacity to Consent 23
4.3.4 NIH Employees 23
4.4 Clinical Procedures and Evaluation 23
4.4.1 Recruitment and Retention Strategies 24
4.4.2 Costs 24
4.4.3 Compensation 24
4.4.4 Screening 24
4.4.5 Study Schedule 25
4.4.6 Enrollment and Day 0 25
4.4.7 Birth Control and Pregnancy 25
4.4.8 Vaccine Administration 26
4.4.9 Post-Product Administration Follow-Up 26
4.4.10 Solicited Adverse Events (Reactogenicity) 26
4.4.11 Follow-up through End of Study 27
4.4.12 Blood Sample Collection 27

4.4.13 Apheresis 27
4.4.14 Apheresis Eligibility Criteria 28
4.4.15 Concomitant Medications 29
4.5 Criteria for Dose Escalation 29
4.6 Group 4 Dose Selection 30
4.7 Criteria for Discontinuing Protocol Participation 30
4.8 Discontinuing Second Vaccination 30
4.9 Criteria for Pausing and Resuming the Study 31
4.9.1 Plan for Pausing the Study 31
4.9.2 Plan for Review of Pauses and Resuming the Study 32
5. SAFETY AND ADVERSE EVENT REPORTING 33
5.1 Adverse Events 33
5.2 Serious Adverse Events 33
5.3 Adverse Event Reporting to the IND Sponsor 34
5.4 Adverse Events of Special Interest (AESI) 34
5.4.1 Anaphylaxis 35
5.4.2 Myocarditis and Pericarditis 35
5.5 Medically Attended Adverse Events (MAAEs) 35
5.6 Reporting of Serious Adverse Events to DMID Pharmacovigilance 35
5.7 Reporting of Pregnancy 36
5.8 IND Sponsor Reporting to the FDA 36
5.9 Reporting to the Institutional Review Board 37
5.9.1 Unanticipated Problem 37
5.9.2 Non-Compliance 38
5.9.3 Protocol Deviation 38
5.9.4 Death 39
5.9.5 New Information 39
5.9.6 Suspension or Termination of Research Activities 39
5.9.7 Expedited Reporting to the IRB 39
5.9.8 Annual Reporting to the IRB 39
6. STATISTICAL CONSIDERATIONS 40
6.1 Overview 40
6.2 Sample Size and Accrual 40
6.3 Endpoints 40
6.3.1 Primary Endpoints: Safety 40
6.3.2 Secondary Endpoints: Immunogenicity 40
6.3.3 Exploratory Endpoints: Immunogenicity 40
6.3.4 Sample Size Consideration for Safety 40
6.3.5 Sample Size Consideration for Immunogenicity 42
6.3.6 Sample Size Consideration for Comparison 42
6.4 Statistical Analysis 42
6.4.1 Analysis Variables 42
6.4.2 Baseline Demographics 42

6.4.3 Safety Analysis 43
6.4.3.1 Solicited Reactogenicity 43
6.4.3.2 Adverse Events 43
6.4.3.3 Local Laboratory Values 43
6.4.4 Immunogenicity Analysis 43
6.4.5 Missing Data 43
6.4.6 Interim Analyses 44

7. PHARMACY AND VACCINE ADMINISTRATION PROCEDURES 45

7.1 mRNA-1215 Vaccine, Pharmaceutical Formulation 45
7.2 Vaccine Storage 45
7.2.1 Handling Information 45
7.2.2 Temperature Excursions 45
7.3 Preparation of mRNA-1215 for Administration 46
7.3.1 Dose Preparation 46
7.4 Administration of mRNA-1215 Injection 47
7.5 Study Product Accountability 47
7.5.1 Documentation 47
7.5.2 Disposition 47

8. HUMAN SUBJECT PROTECTIONS AND ETHICAL OBLIGATIONS 48

8.1 Institutional Review Board 48
8.2 Informed Consent 48
8.3 Study Discontinuation and Closure 48
8.4 Confidentiality and Privacy 49
8.5 Risks and Benefits Assessment 50
8.5.1 Risks of mRNA-1215 50
8.5.2 Risks of Specimen Collection 50
8.5.3 Risks of mRNA-1215 for the Fetus or Nursing Infant 51
8.5.4 Risks of New Diagnoses 51
8.5.5 Potential Benefits 51
8.5.6 Assessment of Potential Risks and Benefits 51
8.6 Plan for Use and Storage of Biological Samples 52
8.6.1 Use of Samples, Specimens and Data 52
8.6.2 Storage and Tracking of Blood Samples and Other Specimens 52
8.6.3 Disposition of Samples, Specimens and Data at Completion of the Protocol 52
8.6.4 Loss or Destruction of Samples, Specimens or Data 53
8.7 Safety Oversight 53
8.7.1 Protocol Safety Review Team 53
8.7.2 Safety Monitoring Committee (SMC) 53

9. ADMINISTRATIVE AND OPERATIONAL OBLIGATIONS 55

9.1 Protocol Amendments 55
9.2 Study Documentation and Storage 55
9.3 Clinical Monitoring 55
9.4 Data Collection and Data Sharing 56

9.4.1 Data Collection 56
9.4.2 Source Documents 56
9.4.3 Data Sharing Plan 56
9.5 Quality Assurance and Quality Control 56
9.6 Language 56
9.7 Research-Related Injuries 57
10. REFERENCES 58
APPENDIX I: SCHEDULE OF EVALUATIONS 61
APPENDIX II: ASSESSMENT OF RELATIONSHIP TO VACCINE AND GRADING SEVERITY OF ADVERSE EVENTS 66
APPENDIX III: ADVERSE EVENTS OF SPECIAL INTEREST TERMS 74

LIST OF TABLES

Table 1: VRC 322/DMID 21-0016 Study Schema 20
Table 2: Probability of Observing Events under Different Scenarios within a Group (n=10) 41
Table 3: 95% Confidence Intervals for True Rate under Possible Observed Number of Events . 41
Table 4: Minimum Event Rate That Can Be Detected With 80% Or 90% Power with Sample Size n=10 per Arm 42
Table 5: mRNA-1215 Injection Dose Preparation Schema 47
Table 6: AESI Categories 75
Table 7: Case Definitions of Probable and Confirmed Myocarditis, Pericarditis, and Myopericarditis 76

ABBREVIATIONS

Abbreviation	Definition
ADA	Anti-Drug Antibody
AE	Adverse event
AoU	Assessment of Understanding
ALP	Alkaline phosphatase
ALT	Alanine aminotransferase
AST	Aspartate aminotransferase
BD	Bangladesh strain
CBC	Complete blood count
CDC	Centers for Disease Control and Prevention
CEPI	Coalition for Epidemic Preparedness Innovations
cGMP	current Good Manufacturing Practices
CMP	Clinical Monitoring Plan
CTP	Clinical Trials Program
DMID	Division of Microbiology and Infectious Diseases
FDA	Food and Drug Administration
GCP	Good Clinical Practice
GMT	Geometric Mean Titer
ICF	Informed Consent Form
ICH	International Council for Harmonisation
IM	Intramuscular Injection
IND	Investigational New Drug Application
IND SAR	Investigational New Drug Application Sponsor's Authorized Representative
IRB	Institutional Review Board
IUD	Intrauterine device
LNP	Lipid Nanoparticle
MAAE	Medically attended adverse event
MedDRA	Medical Dictionary for Regulatory Activities
MM	Medical Monitor
MY	Malaysian strain
NaCl	Sodium Chloride
NHP	Non-human primate
NIAID	National Institute of Allergy and Infectious Diseases
NIH	National Institutes of Health
NIH CC	National Institutes of Health Clinical Center
NiV	Nipah virus
PBMC	Peripheral blood mononuclear cell
PCR	Polymerase Chain Reaction
PI	Principal Investigator
PSRT	Protocol Safety Review Team
QA	Quality Assurance

Abbreviation	Definition
SAE	Serious adverse event
SC	Subcutaneous
SMC	Safety Monitoring Committee
SUSAR	Serious and unexpected suspected adverse reaction
U.S.	United States
ULN	Upper limit of normal
VEC	Vaccine Evaluation Clinic
VRC	Vaccine Research Center
VIP	Vaccine Immunology Program
VRC	Vaccine Research Center
WBC	White blood cells
WHO	World Health Organization

PRÈCIS

Title: **VRC 322/DMID 21-0016**: A Phase I, Dose Escalation, Open-Label Clinical Trial to Evaluate Safety, Tolerability, and Immunogenicity of a Nipah Virus (NiV) mRNA Vaccine, mRNA-1215, in Healthy Adults

Design: This Phase I, dose escalation, open label clinical trial is the first study of mRNA-1215 in healthy adults to evaluate the safety, tolerability, and immunogenicity of a Nipah virus (NiV) mRNA vaccine. The hypotheses are that the vaccine will be safe, tolerable, and will elicit an immune response in healthy adults.

Study Product: mRNA-1215 is a novel mRNA vaccine that encodes for the secreted prefusion stabilized F component covalently linked to G monomer (pre-F/G) of Malaysian strain NiV, resulting in a post-expression trimerization. mRNA-1215 was co-developed by the Vaccine Research Center (VRC), National Institute of Allergy and Infectious Disease (NIAID) and ModernaTX, Inc, and manufactured by ModernaTX.

Subjects: Healthy adults, 18 to 60 years of age.

Plan: Subjects will be enrolled at the NIH Clinical Center and will receive mRNA-1215 via intramuscular (IM) injection by needle and syringe into the deltoid muscle. A dose escalation safety evaluation will occur to ensure the safety data support proceeding to the higher dose group. The mRNA-1215 vaccine dose for Group 4 was selected based on interim analysis of safety and immunogenicity data from Groups 1-3. Subjects will be evaluated for safety and immune responses through clinical observation and blood collection at specified timepoints throughout the study. The study schema is as follows:

Study Schema				
**Group**	**Subjects**	**Dose/Route**	**Day 0**	**Week 4**
1	10	25 mcg IM	X	X
2	10	50 mcg IM	X	X
3	10	100 mcg IM	X	X
4	10	10 mcg IM	X	X
**Total**	[1]40	[1]Enrollment up to 50 subjects is permitted in case additional evaluations are required for safety or immunogenicity.		

Duration: Subjects will be evaluated for safety and immune responses throughout the study for 52 weeks following last product administration.

1. INTRODUCTION

The Nipah virus (NiV) is a zoonotic single-stranded, negative sense RNA virus belonging to the *Paramxyxoviridae* family, genus *Henipavirus,* which also includes Hendra virus (HeV) and Cedar virus (CedPV). NiV and HeV cause fatal disease in humans while CedPV has not been found to be pathogenic in any animal [1]. Two different strains of NiV have been identified, the Malaysian (MY) and the Bangladesh (BD) strains. The two strains are approximately 92% identical in sequence but appear to be significantly different in their pathogenicity and transmissibility [2]. The MY strain of NiV is primarily encephalitic with no documented cases of person-to-person transmission [3]. While most cases result from zoonotic transmission, the BD strain of NiV can also spread person-to-person via respiratory (droplet) transmission [4-9]. Research in non-human primates (NHP) and ferrets has shown there is a more rapid onset of productive infection and higher levels of virus replication in respiratory tract tissues observed after NiV-BD infection compared to NiV-MY infection[10-12]. In addition, NiV-BD infection in humans resulted in a higher mortality rate compared to NiV-MY. This difference could be attributed to inadequate regional medical care[13]. Infection in humans is neurotropic, and patients often develop fatal encephalitis [14-19]. NiV can cause illness in horses and other domestic animals such as dogs, cats and goats which increases the probability of zoonotic transmission from intermediate hosts [14, 20-27].

The first outbreak of NiV infection occurred in the Malaysian peninsula and was a major epidemic of fatal viral encephalitis in people and respiratory and neurological illness in pigs from September 1998 to June 1999. The initial outbreak crippled the lucrative swine industry in Malaysia in less than a year. After the initial outbreak in Malaysia and Singapore, a second NiV outbreak in Bangladesh occurred in 2001 and the Indo-Bangladesh outbreaks have occurred nearly annually since then [15, 28]. In May 2018, the first NiV outbreak in southern India (Kerala) occurred in which there were 23 cases of NiV infection of which 21 resulted in death [6]. The Pteropus genus of large fruit bats have been identified as the natural reservoir of *Henipaviruses* and the known vector of NiV [29-31]. The transmission of NiV infection from bats to humans can occur via multiple routes; human ingestion of fresh date palm sap contaminated by the urine, saliva or feces of Pteropid bats; human or animal consumption of fruit partially eaten by the bat; or human contact with infected domestic animals [8, 14, 30].

The characteristics of NiV that enhance the risk of this virus causing a global pandemic are 1) NiV is highly pathogenic to a broad range of mammals; 2) NiV is capable of person-to-person transmission; 3) high mortality rate associated with infection. A licensed vaccine or therapeutic is not available for NiV, even though NiV is classified as a Biological Safety Level 4 (BSL 4) pathogen, considered a pandemic threat and listed as a high priority pathogen for intervention development by the World Health Organization (WHO), Centers for Disease Control and Prevention (CDC), and the Coalition for Epidemic Preparedness Innovations (CEPI) [32].

1.1 Study Rationale

1.1.1 Rationale for Development of NiV mRNA-1215 Vaccine

ModernaTX, Inc. developed a rapid response, proprietary messenger RNA (mRNA)-based vaccine platform. This is based on the principle and observations that antigens can be produced

in vivo by delivery and uptake of the corresponding mRNA by cells. ModernaTX, Inc. in collaboration with the Vaccine Research Center (VRC) used its mRNA-based technology to develop a novel mRNA vaccine that encodes for the secreted prefusion stabilized F component covalently linked to G monomer (pre-F/G) of NiV, resulting in a post-expression trimerization.

The VRC evaluated NiV vaccine antigen design options including the fusion glycoprotein (F) and the major attachment glycoprotein (G). A stabilized prefusion F (pre-F), multimeric G constructs, and chimeric proteins containing both pre-F and G were developed as protein subunit candidate vaccines. The proteins were evaluated for antigenicity and structural integrity using kinetic binding assays, electron microscopy, and other biophysical properties.

Immunogenicity of the vaccine antigens was evaluated in mice. The stabilized pre-F trimer and hexameric G immunogens both induced serum neutralizing activity in mice, while the post-F trimer immunogen did not elicit neutralizing activity. The pre-F trimer covalently linked to G monomer (pre-F/G) induced potent neutralizing antibody activity and elicited responses to the greatest diversity of antigenic sites. The pre-F/G molecule is the lead candidate for clinical development [33, 34].

1.1.2 Rationale for mRNA-1215 Vaccine Dose and Regimen

Phase 1 to 3 clinical trial data showed that SARS-CoV-2 mRNA vaccines administered at doses ranging from 25 mcg to 100 mcg were safe and well tolerated [35-38]. The mRNA-1215 vaccine dose levels chosen to be clinically tested in this study are supported by published and available data of mRNA vaccines in humans as well as preclinical immunogenicity studies post administration of SARS-CoV-2 mRNA vaccines (Moderna, mRNA-1273 vaccine, and BNT162b2 vaccine, BioNTech/Pfizer) in mice and non-human primates (NHP). SARS-CoV-2 specific antibodies were induced by doses ranging from 0.2-100 mcg [39-42]. Analyses of antibody responses elicited by different vaccine doses indicated that the mRNA vaccines induced a dose-dependent SARS-CoV-2-specific antibody response upon priming that was enhanced by a booster immunization [39-41, 43, 44]. The SARS-CoV-2 mRNA vaccines were able to neutralize the virus in vitro as measured by pseudovirus and SARS-CoV-2 neutralization assays, and antibody levels were sustained for two months or more post-immunization.

A vaccination strategy to achieve protective immunity in most recipients with a single vaccination would be desirable in an outbreak setting. Vaccination strategies that achieve durable protective immunity would be desirable for populations in areas of the world where outbreaks occur sporadically, and these strategies are to be further investigated in future studies in endemic populations.

As indicated in the interim immunogenicity analysis for this study (Section 1.3.1), subjects in Groups 1-3 had a robust response to the vaccine and there was no significant difference in the binding or neutralizing antibody titers to pre-F or G proteins 2 weeks after the second dose between the dose groups. Therefore, the decision was made to test a lower dose of 10 mcg in Group 4 and use the same prime-boost interval of 4 weeks to enable a comparison between the doses (Section 4.6).

1.2 Background

1.2.1 Previous Human Experience with mRNA-based Vaccines

This is the first clinical study to evaluate the mRNA-1215 vaccine, therefore there is no previous human experience with this investigational vaccine product prior to this trial.

Two mRNA-based SARS-CoV-2 vaccines, Moderna's mRNA-1273 vaccine and Pfizer/BioNTech's BNT162b2 vaccine, both produce a stabilized version of the spike glycoprotein [45], show >94% efficacy against symptomatic COVID-19 in interim Phase 3 analyses [35, 36], and are safe and effective. These vaccines are currently being administered to millions of people globally, and the most common adverse events are transient local and systemic reactions. Anaphylactic-like reactions have rarely occurred among recipients of mRNA COVID-19 vaccines. Myocarditis and pericarditis have been reported rarely following vaccination with mRNA COVID-19 Vaccines. These cases are generally mild, occurring a few days to up to a week after the vaccination and are more common after the second dose.

1.2.2 Preclinical Studies with mRNA-1215 Vaccine

In a non-GLP rat immunogenicity study, mRNA-1215 was administered to Sprague Dawley rats via IM injection on Days 1 and 22 at levels of 30, 60, and 100 mcg/dose. Each dose level elicited strong binding and neutralizing antibodies in rats 13 days post-second dose (Day 35). mRNA-1215 was well tolerated up to the high dose of 100 mcg/dose. There were no mRNA-1215 related mortalities, changes in body weight or weight gain. Clinical observations, clinical chemistry, and transient injection site reactions were consistent with a mild-to-moderate inflammatory response, which is anticipated for a vaccine drug product.

Refer to the Investigator's Brochure for additional information on the pre-clinical studies with mRNA-1215.

1.2.3 Human Experience with mRNA-1215 Vaccine

As of March 29, 2023, 30 subjects have been enrolled in Groups 1-3 of this study, completed the product administration schedule per protocol and remain active on the study. The first subject enrollment and study product administration occurred on July 11, 2022. The last Group 3 enrollment occurred on December 27, 2022, and the last Group 3 study product administration occurred on January 24, 2023. No Serious Adverse Events (SAEs) occurred and no stopping or pausing rules were met at any time during this period.

Data collection and monitoring are in progress at the time of this report, and the data can't be considered final. Of the 30 subjects enrolled, 28 (93.3%) reported at least one or more local solicited AEs with the maximum severity being severe for 1 (3.3%) subject, moderate for 1 (3.3%) subject, and mild for 26 (86.7%) subjects. The most common local symptom was mild to moderate pain/tenderness at the injection site reported by 28 (93.3%) subjects, with one (3.3%) subject in Group 3 (100 mcg) reporting moderate and 27 (90.0%) subjects reporting mild severity.

In Group 3 (100 mcg), one subject reported mild swelling (4.0 cm) and mild redness (4.5 cm) 1 day after the second dose that resolved in 1 and 3 days, respectively. One subject reported

severe redness (10.5 cm) 2 days after the first dose, and mild redness (4.5 cm) 2 days after the boost, and both events resolved the same day.

Two (6.7%) subjects, one in Group 1 (25 mcg) and one in Group 3 (100 mcg), reported mild pruritis after the second dose that resolved the same day. No axillary lymphadenopathy has been reported.

All local solicited reactogenicity symptoms resolved within the 7-day reactogenicity collection period without residual effects except for one event of mild/pain tenderness which started one day after product administration and resolved 7 days after onset.

Of the 30 subjects enrolled, 18 (60.0%) reported at least one or more systemic solicited AEs with the maximum severity being severe for 1 (3.3%) subject, moderate for 4 (13.3%) subjects, and mild for 13 (43.3%) subjects. The most common systemic symptom was mild to moderate malaise reported by 16 (53.3%) subjects, with two (6.7%) subjects in Group 3 (100 mcg) reporting moderate and 14 (46.7%) subjects reporting mild severity [2 in Group 1 (25 mcg), 6 each in Group 2 (50 mcg) and Group 3 (100mcg)]. Headaches were reported by 14 (46.7%) subjects, with 1 (3.3%) subject in Group 2 (50 mcg) reporting moderate and 13 (43.3%) subjects reporting mild maximum severity (4 in Group 1, 3 in Group 2, and 6 in Group 3). Myalgia was reported by 11 (36.7%) subjects: 1 (3.3%) subject in Group 3 (100 mcg) reported moderate and 10 (33.3%) reported mild severity (1 in Group 1, 4 in Group 2, and 5 in Group 3). Mild to moderate chills were reported by 6 (20.0%) subjects, with 3 (10.0%) reporting mild (1 in each group) and 3 (10.0%) in Group 3 (100 mcg) reporting moderate severity. Mild to moderate nausea was reported by 6 (20.0%) subjects, with 5 (16.7%) reporting mild (2 in Group 1, 2 in Group 2, and 1 in Group 3) and 1 (3.3%) in Group 3 (100 mcg) reporting moderate severity. Mild joint pain was reported by 5 (16.7%) subjects (1 in Group 1, 1 in Group 2, and 3 in Group 3). An elevated temperature was reported by 2 (6.7%) subjects in Group 3 (100 mcg): 1 (3.3%) subject reported severe and 1 (3.3%) reported mild severity. The severe temperature elevation occurred in a Group 3 subject on the day of the 2nd study vaccination and resolved the same day.

All solicited systemic reactogenicity symptoms resolved within the 7-day reactogenicity collection period without residual effects with the exception of malaise and headache events reported by 4 (13.3%) subjects: 1 (3.3%) Group 2 (50 mcg) subject reported malaise after the 2nd study vaccination that resolved 10 days after onset; 1 (3.3%) Group 2 (50 mcg) subject reported a headache of 8 day duration after the 1st study vaccination and headache and malaise of 7 day duration each that started one day after the 2nd study vaccination; two (6.7%) Group 3 (100 mcg) subjects reported malaise, 1 (3.3%) of 11 day duration after the 1st study vaccination, and 1 (3.3%) of 1 day duration that started on day 7 after the 2nd study vaccination.

Sixteen (16/30, 53.3%) subjects experienced one or more unsolicited AEs. The maximum severity was severe for one (3.3%), moderate for 7 (23.3%) and mild for 8 (26.7%) subjects. The most common unsolicited AE was anemia in 5 (16.7%) subjects. All events were evaluated as unrelated to study product and occurred most likely due to frequent blood draws required for this study. A severe (Grade 3) muscle strain occurred in a Group 2 (50 mcg) subject one day after the 1st study vaccination. The subject was lifting heavy objects at work and was evaluated in the emergency room. This event was classified as a Medically Attended Adverse Event (MAAE) but was not an SAE or an Adverse Event of Special Interest (AESI), and it was evaluated as not related to study product.

Six (20.0%) subjects had the following events that were evaluated as related to study product: One (3.3%) Group 3 (100 mcg) subject had a moderate AE of dizziness after the 2nd vaccination while sitting and standing that interfered with usual activities and resolved 1 day after product administration. One (3.3%) Group 3 (100 mcg) subject developed a moderate urticarial rash occurring on the torso, extremities, hands and feet which began 12 days after the 1st study vaccination. Because the subject did not inform the study team about the hives at that time, the 2nd study vaccine was administered and exacerbated the hives. This AE is ongoing, but significantly improved with sporadic hives treated as needed with antihistamines; it is classified as a MAAE and a new onset chronic medical condition. It was assessed as related to study product.

One (3.3%) Group 3 (100 mcg) subject had mild hypertension 30 minutes after the 1st study vaccination that resolved 7 days after onset, and three (10.0%) subjects had mild leukopenia, which self-resolved without residual effects as noted below:

- One Group 3 (100 mcg) subject had leukopenia (WBC 3.35 K/mcL) 7 days after the 2nd study vaccination that resolved 7 days after onset,
- One Group 2 (50 mcg) subject had leukopenia (WBC 3.24 K/mcL) 7 days after the 1st study vaccination that resolved 7 days after onset, and
- One Group 3 (100 mcg) subject had leukopenia (WBC 3.01 K/mcL; ALC 0.94 K/mcL) 7 days after the 1st study vaccination that resolved 22 days after onset.

No AESIs have been reported.

Conclusions: Study product administrations were overall well tolerated. No product related serious adverse events occurred. Solicited local reactogenicity was predominantly mild pain/tenderness with similar frequencies between groups and after each dose. Solicited systemic reactogenicity occurred more frequently after the second dose and symptoms were experienced more frequently in the 100 mcg dose group. Solicited systemic events graded as moderate or severe occurred primarily in the 100 mcg dose group. All related, unsolicited events have resolved, except for urticaria in one participant in the 100 mcg dose group.

Based on the data collected in this study so far, no safety concerns have emerged from the study of mRNA-1215 Nipah vaccine.

1.3 Laboratory Assessment of mRNA-1215 Vaccine Immunogenicity

In this study, specimens to evaluate immunogenicity will be collected at baseline and at specified time points as indicated in APPENDIX I, Schedule of Evaluations. The primary immunogenicity time point is two weeks after last product administration. NiV-specific humoral immune responses will be assessed. Additional assessments of specific antibody responses may be conducted on stored samples obtained throughout the study.

Exploratory evaluations may include the detection of antibodies (Ab) to NiV by neutralization and functional serological assays, and exploratory B and T cell assays. To support development of diagnostics, therapeutics and future vaccines, optional leukapheresis will be offered to subjects at two weeks post second vaccination.

Research samples will be processed by the Vaccine Immunology Program (VIP) in Gaithersburg, MD, which will also perform some of the immunogenicity assays. Some immunogenicity assays will be performed by the VRC laboratories in Bethesda, MD, or by approved contract laboratories or research collaborators.

Results from this study are expected to contribute to the fund of knowledge needed for the development of a NiV mRNA vaccine candidate as well as to show proof-of-concept for safety and elicitation of antibody responses induced by a NiV mRNA vaccine.

1.3.1 Interim Evaluation of the mRNA-1215 Vaccine Immunogenicity

Serum samples collected at 2 weeks post boost (week 6, visit 07) were used for interim evaluation of vaccine immunogenicity to inform Group 4 dose selection. Responses to pre-F and mono-G were evaluated as described in Section 1.3.

Subjects with evaluable samples (N=29) all had a robust response to the vaccine, and there was no significant difference in the binding antibody titers to pre-F or G proteins 2 weeks after the second dose between dose groups. Similarly, there were no differences in neutralizing antibody titers at this timepoint between the dose groups.

In addition, immune responses at 12 weeks after the boost (week 16, visit 09) were evaluated. The binding and neutralizing antibody titers at week 16 have declined in comparison to the immune responses at week 6, however, no significant differences in the week 6 to week 16 decline were found between the dose groups.

2. STUDY PRODUCTS

2.1 mRNA-1215 Vaccine

The investigational mRNA-1215 vaccine is a lipid nanoparticle (LNP) dispersion that contains an mRNA encoding for the secreted prefusion stabilized trimeric F component covalently linked to G monomer (pre-F/G) of Malaysian strain NiV, resulting in a post-expression trimerization. It is provided as a sterile liquid for intramuscular (IM) injection, with white to off white dispersion in appearance. A pharmaceutical composition of the investigational product is described in Section 7.1.

2.2 Diluent: 0.9% NaCl for injection, USP

0.9% NaCl for Injection, USP or normal saline is a sterile, nonpyrogenic, isotonic solution that will be used as a diluent to dilute the vaccine (mRNA-1215) to the desired concentration.

3. STUDY OBJECTIVES

3.1 Primary Objectives

- To evaluate the safety and tolerability of a 2-dose vaccination regimen of mRNA-1215 at doses of 10 mcg, 25 mcg, 50 mcg or 100 mcg administered IM, given at a 4-week interval

3.2 Secondary Objectives

- To evaluate antibody responses to the mRNA-1215 vaccine at doses of 10 mcg, 25 mcg, 50 mcg or 100 mcg at 2 weeks after last product administration

3.3 Exploratory Objectives

- To evaluate the specificity and functionality of mRNA-1215 vaccine-induced antibodies and the immune response in all study groups at various timepoints throughout the study
- To evaluate the frequency, magnitude, phenotype, and specificity of B-cell, T-cell, and antibody responses at various time points throughout the study

4. SUBJECT POPULATION AND CLINICAL PROCEDURES

4.1 Overall Study Design

This is a Phase I, dose escalation, open-label clinical trial to evaluate dose, safety, tolerability and immunogenicity of mRNA-1215 vaccine in healthy adults. The study schema is shown in **Table 1**. The hypotheses are that the vaccine is safe, tolerable and will induce an antibody response to the Nipah Virus (NiV). The study will be conducted by the VRC Clinical Trials Program at a single site (Vaccine Evaluation Clinic, VEC) at the NIH Clinical Center (NIH CC), Bethesda, MD.

Table 1: VRC 322/DMID 21-0016 Study Schema

Study Schema				
**Group**	**Subjects**	**Dose/Route**	**Day 0**	**Week 4**
1	10	25 mcg IM	X	X
2	10	50 mcg IM	X	X
3	10	100 mcg IM	X	X
4	10	[1]10 mcg IM	X	X
**Total**	[1]40	[1]Enrollment up to 50 subjects is permitted in case additional evaluations are required for safety or immunogenicity.		

The study will have staged enrollment with required interim safety reviews, as described in Section 4.5, Criteria for Dose Escalation. The study opened with enrollments in Group 1. The dose for Group 4 was chosen based on interim analysis of safety and immunogenicity data from Groups 1-3 (Section 4.6). Subjects will be evaluated for safety and immune responses throughout the study for 52 weeks following last product administration.

4.2 Study Population

All inclusion and exclusion criteria must be evaluated for eligibility.

4.2.1 Inclusion Criteria

A subject must meet all of the following criteria:

1. Healthy adults between the ages of 18-60 years inclusive.
2. Based on history and physical examination, in good general health and without history of any of the conditions listed in the exclusion criteria.
3. Able and willing to complete the informed consent process.
4. Available for clinic visits for 52 weeks after last product administration.
5. Able to provide proof of identity to the satisfaction of the study clinician completing the enrollment process.
6. Physical examination and laboratory results without clinically significant findings and a Body Mass Index (BMI) of 18 to 35 within the 56 days before enrollment.

Laboratory Criteria within 56 days before enrollment

7. White blood cells (WBC) and differential within institutional normal range or accompanied by the site Principal Investigator (PI) or designee approval.
8. Total lymphocyte count ≥ 800 cells/µL.
9. Platelets = 125,000 – 500,000 cells/µL.
10. Hemoglobin within institutional normal range or accompanied by the PI or designee approval.
11. Alanine aminotransferase (ALT) ≤ 1.25 x institutional upper limit of normal (ULN).
12. Aspartate aminotransferase (AST) ≤ 1.25 x institutional ULN.
13. Alkaline phosphatase (ALP) <1.1 x institutional ULN.
14. Total bilirubin within institutional normal range or accompanied by the PI or designee approval.
15. Serum creatinine ≤ 1.1 x institutional ULN.
16. Negative for HIV infection by an FDA-approved method of detection

***Criteria applicable to women of childbearing potential*:**

17. Negative beta-human chorionic gonadotropin (β-HCG) pregnancy test (urine or serum) on the day of enrollment.
18. Agrees to use an effective means of birth control from at least 21 days prior to enrollment through the end of the study.

4.2.2 Exclusion Criteria

A subject will be excluded if one or more of the following conditions apply:

1. Breast-feeding or planning to become pregnant during the study.
2. More than 10 days of systemic immunosuppressive medications or cytotoxic medications within the 4 weeks prior to enrollment or any within the 14 days prior to enrollment.
3. Blood products within 16 weeks prior to enrollment.
4. Any vaccine, including COVID-19 vaccines, received within 4 weeks prior to enrollment.
5. Investigational research agents within 4 weeks prior to enrollment or planning to receive investigational products while on the study.
6. Current allergy treatment with allergen immunotherapy with antigen injections, unless on maintenance schedule.
7. Current anti-TB prophylaxis or therapy.
8. Known immediate hypersensitivity to any component of the study product, including polyethylene glycol (PEG).

9. Confirmed past NiV infection, prior residence in (>6 months), or planned travel for any length of time during the study to countries where NiV infection is endemic, eg. Bangladesh, India, Philippines.

Subject has a history of any of the following clinically significant conditions:

10. Serious reactions to vaccines that preclude receipt of the study vaccination, including allergic reaction (anaphylaxis, urticaria or allergic reaction requiring medical intervention) to SARS-CoV-2 mRNA vaccines, as determined by the investigator
11. History of myocarditis and/or pericarditis
12. Hereditary angioedema, acquired angioedema, or idiopathic forms of angioedema
13. Asthma that is not well controlled
14. Diabetes mellitus (type I or II), with the exception of gestational diabetes
15. Thyroid disease that is not well controlled
16. Idiopathic urticaria within the past year
17. Autoimmune disease or immunodeficiency
18. Hypertension that is not well controlled
19. Bleeding disorder diagnosed by a doctor (e.g. factor deficiency, coagulopathy, or platelet disorder requiring special precautions) or significant bruising or bleeding difficulties with IM injections or blood draws
20. Malignancy that is active or history of malignancy that is likely to recur during the period of the study
21. Seizure disorder other than 1) febrile seizures, 2) seizures secondary to alcohol withdrawal more than 3 years ago, or 3) seizures that have not required treatment within the last 3 years.
22. Asplenia, functional asplenia or any condition resulting in the absence or removal of the spleen
23. Guillain-Barré Syndrome
24. Any medical, psychiatric, social condition, occupational reason or other responsibility that, in the judgment of the investigator, is a contraindication to protocol participation or impairs a subject's ability to give informed consent, including but not limited to clinically significant forms of: infectious diseases, drug or alcohol abuse, autoimmune diseases, psychiatric disorders, or heart disease.

4.3 Inclusion of Vulnerable Subjects

4.3.1 Pregnant Women

This is a first-in-human trial in healthy subjects, ≥18 years of age. Because the effects of the vaccine on the fetus are not known, pregnant women will not be eligible for the trial. Women of childbearing potential must utilize a highly effective method of contraception and will be

required to have a negative urine or serum pregnancy test within 24 hours prior to each vaccination.

4.3.2 Children

Children are not eligible to participate in this clinical trial because the investigational vaccine has not been previously evaluated in adults. If the product is assessed as safe and immunogenic, other protocols designed for children may be conducted in the future.

4.3.3 Adult Subjects who Lack the Capacity to Consent

Adults who are unable to provide initial informed consent are excluded to enroll. Also, adults who permanently lose the capacity to provide on-going consent after initial consent and during the study will be discontinued from protocol participation as it is described in Section 4.7.

4.3.4 NIH Employees

NIH employees and members of their immediate families may participate in this protocol. Neither participation nor refusal to participate will have an effect, either beneficial or adverse, on the participant's employment or work situation. We will follow the Guidelines for the Inclusion of Employees in NIH Research Studies and will give each employee a copy of the "NIH FAQs for NIH Staff Who are Considering Participation in NIH Research" published by Office of Human Research Subjects Protections on Research Involving NIH Staff as Subjects, Policy 404.

For NIH employee subjects, consent will be obtained by an individual who is independent of the employee's team.

If an NIH staff member seeks to enroll in research taking place within their own work unit or conducted by any of their supervisors, the employee will be:

- Informed that neither participation nor refusal to participate as a research subject will have an effect, either beneficial or adverse, on the subject's employment, training or position at the NIH,
- When possible, consent should be obtained by an individual in a non-supervisory relationship with the subject, and
- When consent is conducted, a third party (e.g. a consent monitor) will be included through the Bioethics Consultation Service or another party independent of the research team or, if a consent monitor is not available, the consent process will be observed by another qualified investigator on the study who is independent of the NIH staff member's work unit and not a supervisor to the NIH staff member. If no such person exists, consent observation may be performed by any qualified investigator on the study. Protocol study staff will be trained on obtaining potentially sensitive and private information from co-workers or subordinates.

4.4 Clinical Procedures and Evaluation

Evaluation of mRNA-1215 vaccine will include laboratory tests, medical history, physical assessment by clinicians, and subject self-assessment recorded on a diary card for 7 days after

injection. The schedule of study evaluations is described in this section and shown in table format in APPENDIX I.

4.4.1 Recruitment and Retention Strategies

Study enrollments will be conducted at the NIH Clinical Center. Study subjects will be recruited through the VRC's screening protocol, VRC 500 (NCT 01375530). The on-site and off-site Institutional Review Board (IRB)-approved advertising will be implemented. Per a recruitment plan described in the VRC 500 protocol, efforts will be made to include women and minorities in proportions similar to that of the community from which they are recruited.

4.4.2 Costs

There are no costs to subjects for their participation in this trial.

4.4.3 Compensation

Subjects will be compensated for time and inconvenience in accordance with the standards for compensation of the NIH Clinical Research Volunteer Program. The compensation per visit will be $315 for the visits that include the administration of the study product and $200 for clinic visits that include a blood draw. The compensation for any clinic visit that does not include any specimen collection will be $85. The compensation for timely completion for all 7 days of the electronic diary card will be $25. Compensation will be $285 for apheresis, if performed. The total compensation for the subject is based on the number of study clinic visits completed, performance of optional research blood collections and timely submission of electronic diary cards.

The total amount of compensation ($2,830 –$2,965) varies depending upon number and type of visits completed. Compensation may also vary depending upon participation in the optional apheresis and completion of electronic diary card. Subjects will usually receive compensation by direct deposit approximately 1 or 2 weeks after each completed visit. Compensation may need to be reported to the Internal Revenue Service (IRS) as taxable income.

4.4.4 Screening

All screening procedures for this study will be completed through the VRC's screening protocol, VRC 500 (NIH 11-I-0164, NCT01375530) used for all VRC IND studies conducted at the NIH Clinical Center. The evaluations and sample collections included in screening are a medical history, physical exam, laboratory tests needed to confirm eligibility, HIV test, and pregnancy test for females of reproductive potential.

If screening evaluations suggest a current concerning health condition or infection, then appropriate clinical laboratory tests may be conducted to evaluate these conditions under screening protocol, VRC 500. Additional assessments of health may be conducted during screening based on clinical judgment. Screening evaluations for specific eligibility criteria (Section 4.2) must be completed within the time interval specified prior to enrollment for the given parameter and may be repeated, as needed, to confirm eligibility. No screening procedures will be done under the protocol VRC 322/DMID-21-0016.

Research blood samples as shown on the SoE may be collected at any time during screening.

Subjects who are not up to date on standard vaccinations may receive these, if available, during their participation in the screening protocol or at a later date during study participation.

The informed consent form (ICF) will be reviewed and counseling related to pregnancy prevention will be provided. As part of the informed consent process, an Assessment of Understanding (AoU) will be completed in association with enrollment into VRC 322/DMID 21-0016. Records will be kept documenting the reason if screened subjects do not enroll.

4.4.5 Study Schedule

The Schedule of Evaluations in APPENDIX I provides details on the study schedule, the permitted windows for completing the visits, and the evaluations to be performed at each visit. The visit schedule is based on intervals of time after study injection. The clinicians will discuss the target dates and timing of sample collections with each subject before completing enrollment to help ensure that subjects can comply with the projected schedule.

After enrollment, deviations from the visit windows are discouraged and will be recorded as protocol deviations but are permitted at the discretion of the PI (or designee) in the interest of completing the vaccination schedule and obtaining subject safety and immunogenicity evaluations.

If a participant undergoes a treatment with systemic glucocorticoids (e.g., prednisone or other glucocorticoid) or other immunomodulators (other than nonsteroidal anti-inflammatory drugs [NSAIDs]), study vaccination may continue per investigator discretion with the boost dose being preferably delayed to 2 weeks following completion of glucocorticoid treatment. This treatment will be documented as a concomitant medication and any delays in boost vaccinations recorded as a protocol deviation.

Licensed or emergency use authorization (EUA) vaccines should be administered as needed, preferably within 4 weeks before or after each product administration.

4.4.6 Enrollment and Day 0

Day 0 is defined as the day of protocol enrollment and injection for all groups. Protocol-specific eligibility is reviewed on Day 0 as part of the enrollment process, but eligibility evaluations conducted during a screening visit are routinely used for eligibility if evaluations are within the specified window prior to Day 0 as it is described in the Schedule of Evaluations APPENDIX I. However, if clinical assessment on Day 0 suggests that significant changes have occurred since the screening visit, then evaluations done on Day 0 are used for eligibility. Day 0 evaluations and medical history prior to the injection are the baseline for subsequent safety assessments.

The study group assignment in the database will be set up prior to opening the study to accrual. The group assignment is known to the staff and subject before completing the electronic enrollment into the study on Day 0. All subjects that receive mRNA-1215 will be expected to continue with follow-up through the end of the study.

4.4.7 Birth Control and Pregnancy

Women of reproductive potential must agree to the use of an effective method of birth control beginning 21 days prior to enrollment and continuing through end of study.

Acceptable and effective methods of birth control for women of reproductive potential in this study include abstinence (no sex) with male partners, birth control pills or patch, condoms, Medroxyprogesterone acetate (MPA) injection, diaphragm or cervical cap, intrauterine device (IUD), Implant (Nexplanon®), Annovera®, NuvaRing®, partner which has vasectomy.

Pregnancy, if occurred, will be recorded in the study database. These participants will not receive any further study vaccinations and will not have any further research sample collections or procedures. Pregnant participants will continue to be followed for clinical safety and to collect the pregnancy outcome. Any follow-up procedures and/or data collected will be for clinical/safety outcome purposes only. Pregnancy will be reported as described in Section 5.7.

4.4.8 Vaccine Administration

There are 2 study injections to be completed with a 4-week interval. All study injections will be completed according to the assigned group and will be administered IM in the deltoid muscle. Scheduled blood collection must be completed before vaccination.

On the day of and prior to vaccine administration, study subjects will be clinically evaluated and samples will be collected as per Schedule of Evaluations APPENDIX I. A subject who arrives at the clinic with fever or evidence of an acute illness or injury that precludes administration of the vaccine may be rescheduled within the allowed study visit window.

A negative pregnancy test result for women of reproductive potential must be obtained on the day of and prior to each vaccination.

When choosing an arm for injection, clinicians will assess for injury, local skin conditions or tattoos that preclude administration or may interfere with evaluation of the injection site. The non-dominant arm is preferable. Prime and boost vaccinations should preferably be administered in the same arm.

4.4.9 Post-Product Administration Follow-Up

All subjects will be observed for a minimum of 30 minutes following each vaccination. Vital signs (temperature, blood pressure, pulse and respiratory rate) and assessment of local reactogenicity will be performed after product administration.

In keeping with the NIH CC policy and good medical practice, acute medical care will be provided to subjects for any immediate allergic reactions or other injury resulting from participation in this research study.

4.4.10 Solicited Adverse Events (Reactogenicity)

Subjects will be given a “Diary Card” (paper and electronic-based available), a thermometer, and a measuring device. Subjects will use the diary card to record temperature, local and systemic symptoms, and concomitant medications daily for 7 days after each injection. Subjects will be provided training on diary completion and proper usage of the thermometer to measure temperature and the measuring device to measure injection site symptoms. While subjects will be encouraged to use the secure electronic database, they will have the option to complete a paper diary card. When the diary card parameters are recorded directly by the subject in the electronic database, the subject’s electronic record will be the source for these data. When collected on paper, the paper diary card will be the source document. When neither paper nor electronic diary

is available from the subject, the study clinician will document the source of reactogenicity information recorded in the study database.

The solicited reactogenicity signs and symptoms on the diary card will include the following systemic symptoms: generalized symptoms of unusually tired/feeling unwell, fever, muscle aches (other than at injection site), headache, chills, nausea, and joint pain. The following local reactogenicity symptoms will be included: pain/tenderness, redness, swelling, and pruritus at the injection site, and axillary lymphadenopathy ipsilateral to the injection site.

Each day, subjects will also record their highest measured temperature and measurement of the largest diameter of redness and swelling, and highest severity of pruritus at the injection site.

Follow-up on subject well-being will be performed by telephone on the first or second day following vaccination and by clinic visits as shown in the Schedule of Evaluations (APPENDIX I). Subject diaries will be reviewed by a clinician for accuracy and completeness at follow-up visits.

Events following product administration that may require clinical evaluation include but are not limited to rash, urticaria, fever of 38.5˚C (Grade 2) or higher lasting greater than 24 hours, or significant impairment in the activities of daily living. Additionally, other clinical concerns may prompt a study visit based on the judgment of a study clinician.

The following attributes of the solicited AE(s) will be captured in the study database:

- Solicited local or systemic AE that results in a visit to a healthcare practitioner
- Solicited local or systemic AE leading to the participant withdrawing from the study or the participant being withdrawn from the study by the Investigator (AE leading to withdrawal)
- Solicited local or systemic reactogenicity lasting beyond 7 days after injection
- Solicited local or systemic reactogenicity that otherwise meets the definition of an SAE

4.4.11 Follow-up through End of Study

Study follow-up will continue via clinic visits through 52 weeks following the vaccine administration. Refer to Section 4.7 which describes the criteria for discontinuing study participation.

4.4.12 Blood Sample Collection

At intervals throughout the study, blood will be drawn for safety and immunologic assays. Blood will be drawn from the arm veins of subjects by standard phlebotomy procedures. The order of blood sample collections is shown in the SoE (APPENDIX I), with samples for safety and primary immunogenicity evaluations collected first, and other research samples as permitted with adequate supplies. Total blood volume drawn from each subject will not exceed the NIH CC guidelines.

4.4.13 Apheresis

Subjects will be offered apheresis as an optional procedure at Visit 07. Apheresis is done in order to collect blood cells of special interest for research purposes. The apheresis procedure will be carried out by trained NIH Department of Transfusion Medicine (DTM) medical staff using automated cell separator devices. All study subjects will be treated according to standard DTM whole blood and apheresis donation policies and procedures. Prior to the scheduling apheresis, the subject must have a venous assessment performed by the DTM staff.

In order to undergo apheresis procedures, a subject must meet the apheresis eligibility criteria as described in Section 4.4.14 and have no medical contraindications, as determined by the DTM staff. A VRC study clinician will complete a checklist for apheresis eligibility before referring a subject for the procedure.

Prior to beginning the apheresis procedure, a study clinician may request in advance that other laboratory samples be collected as needed to monitor the well-being of the subject or if needed by a research laboratory. In addition, for women of reproductive potential, a pregnancy test by blood or urine will be performed by a VRC study clinician within 72 hours prior to the apheresis procedure. Results must be negative to proceed with apheresis.

The Dowling Apheresis Clinic staff at the NIH CC routinely performs a hemoglobin test prior to initiating apheresis, per DTM Apheresis Clinic standard policies. If a subject is found to have a hemoglobin value less than permitted by the Apheresis Clinic, then the apheresis will not be initiated, and the ordering provider will be notified.

In this study, the procedure will require two antecubital venous access sites and will involve processing 1 to 4 liters of whole blood. The expected mononuclear cell yield is approximately 0.5 to 1.0 x 10^9 cells per liter processed, and the apheresis device can process about 2 to 3 liters per hour. Thus, 1 to 2 hours are required to process 1 to 4 liters of blood and obtain about 1 to 4 x 10^9 leukocytes. The packed red cell loss during the procedure is the equivalent of a 6 mL blood draw; this is the volume that will be used for the purposes of calculating cumulative blood draw when apheresis is performed.

During or following an apheresis visit, if there is any concern about the well-being of the subject, the DTM clinic may conduct appropriate medical evaluations by history-taking, physical examination, laboratory tests, and/or other testing.

4.4.14 Apheresis Eligibility Criteria

Subject must meet all of the following criteria:

- Afebrile (temperature < 37.5°C)
- Weight ≥110 pounds
- Adequate bilateral antecubital venous access
- Hemoglobin > 12.5 g/dL for females; > 13.0 g/dL for men
- Platelets > 150,000 cells/µL
- No cardiovascular instability as indicated by: a) history of medically significant cardiac arrhythmia within the last 12 months, or b) ischemic cardiovascular disease within the last 12 months, or c) heart rate outside of the 50 - 100 beats/minute interval (on 3

successive readings), or d) blood pressure greater than 180 mmHg (systolic) or 100 mmHg (diastolic) on 3 successive readings

- No current lung or kidney disease
- No known coagulation disorder
- No sickle cell disease
- No active or chronic hepatitis
- No intravenous injection drug use in the past 5 years
- Not breast feeding
- Negative beta-human chorionic gonadotropin (β-HCG) pregnancy test (urine or serum) performed by a VRC study clinician within 72 hours prior to the apheresis procedure for those women with childbearing potential.

4.4.15 Concomitant Medications

Information about prior and current medications starting 4 weeks prior to enrollment will be collected in the appropriate data collection form. Routine prescription medications in use at the time of enrollment will be entered in the database. Subsequently, the concomitant medications that will be recorded or updated in the database are those associated with an AE requiring expedited reporting or the development of a new chronic condition requiring ongoing medical management. Inclusion of other concomitant medications in the database may also be determined at the discretion of the PI. Otherwise, concomitant medications that include all medications (prescription, over the counter, and supplements) taken throughout the study will be recorded in the subject's study chart and general medical record but will not be recorded in the database. At each study visit following dosing, including telephone calls, subjects will be queried about new concomitant medications and changes to existing medications.

A treatment with systemic glucocorticoids (e.g., prednisone or other glucocorticoid) or other immunomodulators (other than nonsteroidal anti-inflammatory drugs [NSAIDs]) will be recorded in the study database as described in Section 4.4.5.

Receipt of an FDA-approved vaccine at any time during the study will be recorded in the database (clinicians should work with subjects regarding the timing of licensed vaccines relative to study injections, as described in Section 4.4.5).

4.5 Criteria for Dose Escalation

There will be two interim safety data reviews in this study to evaluate safety data for a dose escalation of mRNA-1215 from a 25 mcg dose (Group 1) to a 50 mcg dose (Group 2), and from a 50 mcg to a 100 mcg dose (Group 3). The Protocol Safety Review Team (PSRT) will conduct an interim safety data review before dose escalation may occur. The PSRT must assess the data as showing no significant safety concerns before enrollment of the next dose level may proceed.

Enrollment will begin in Group 1 (25 mcg IM mRNA-1215) with no more than one subject enrolled per day for the first three subjects. When the two weeks post initial vaccination visit for at least 3 subjects in Group 1 is completed, there will be an interim safety review of available

data to determine whether to proceed with the dose escalation to the 50 mcg dose of mRNA-1215.

If the 25 mcg IM dose of mRNA-1215 is assessed as safe, enrollment will begin for Group 2 (50 mcg IM mRNA-1215) with no more than one subject enrolled per day for the first three subjects only. When the two weeks post initial vaccination visit for at least 3 subjects in Group 2 is completed, there will be an interim safety review of available data to determine whether to proceed with the dose escalation to the 100 mcg dose of mRNA-1215 (Group 3).

Consultation with the IRB and FDA, if needed, as per study pause criteria (Section 4.7) will occur if indicated by the review. One outcome of a dose escalation review may be to recommend evaluation of additional subjects at the current dose level and reassess for safety before proceeding to a higher dose level and repeat dosing at the same dose level.

4.6 Group 4 Dose Selection

The Group 4 vaccine dose was selected based on safety and immunogenicity data collected for Groups 1, 2 and 3. Interim safety data collected during the study (Section 1.2.3) and available data on immune responses at 2 and 12 weeks post last vaccination have been reviewed (Section 1.3.1). Additional data collected in other mRNA-based vaccine studies were taken into consideration. The IND Sponsor and the vaccine manufacturer participated in the Group 4 dose selection.

Based on the interim data analysis, the decision was made to use a lower dose of 10 mcg in Group 4 with a prime-boost interval of 4 weeks to enable a comparison between the doses. If the lower dose regimen results in acceptable safety and immunogenicity, this regimen may impact the design of future trials that would be conducted in an endemic country where Nipah virus outbreaks occur.

4.7 Criteria for Discontinuing Protocol Participation

All subjects will be encouraged to remain in the study and to continue follow-up visits to evaluate safety. A subject may be discontinued from protocol participation by the PI or designee for the following reasons:

- Subject voluntarily withdraws
- Subject develops a medical condition that is a contraindication to continuing study participation
- The IND Sponsor or regulatory authority stops the protocol
- The IND Sponsor or PI assesses that is it not in the best interest of the subject to continue participation in the study or that the subject’s compliance with the study is not sufficient.

4.8 Discontinuing Second Vaccination

The PI or designee may also determine that there is a contraindication for administration of the second study vaccination. Subjects who do not get the second injection per protocol will have an

adjusted follow-up schedule and will be asked to continue the study visits and follow-up for safety.

Specific events that will require withdrawal of a subject from the injection schedule include:

1. Pregnancy
2. Grade 2 adverse event assessed as related to a study injection that does not resolve to baseline in time for the next scheduled immunization
3. Grade 3 adverse event assessed as related to a study injection (with the exception that self-limited Grade 3 solicited reactogenicity does not require discontinuation of study injections)
4. Grade 4 adverse event assessed as related to a study injection
5. Immediate hypersensitivity reaction associated with a study injection
6. Intercurrent illness that is not expected to resolve prior to the next scheduled study injection assessed by study clinician to require withdrawal from the injection schedule
7. Repeated failure of participant to comply with protocol requirements
8. The study PI assesses that it is not in the best interest of the subject to continue on the vaccination schedule.

4.9 Criteria for Pausing and Resuming the Study

4.9.1 Plan for Pausing the Study

The PI and Protocol Safety Review Team (PSRT) will closely monitor and analyze study data as it becomes available and will make determinations regarding the presence and severity of AEs. The administration of study injections and new enrollments will be paused and the IND Sponsor will be promptly notified according to the following criteria:

- **One** (or more) subject experiences a **SAE or Grade 4 AE** assessed as related to the vaccine.
- **One** (or more) subject experiences ulceration, abscess or necrosis at the injection site assessed as related to the vaccine.
- **One** (or more) subject experiences laryngospasm, bronchospasm, or anaphylaxis assessed as related to the vaccine.
- **One** (or more) subject experiences myocarditis or pericarditis.
- **Two** (2) or more subjects experience an allergic reaction such as generalized urticaria (defined as occurring at three or more body parts) assessed as related to the vaccine.
- **Three** (3) or more subjects experience a **Grade 3 AE** (unsolicited and/or clinical laboratory abnormality), in the same Preferred Terms based on the medical dictionary for regulatory activities (MedDRA) coding, assessed as related to the vaccine.

4.9.2 Plan for Review of Pauses and Resuming the Study

The IND Sponsor, with participation of the Safety Monitoring Committee (SMC, Section 8.7.2), study PI, PSRT, and vaccine manufacturer, will conduct the review and make the decision to resume, amend or close the study and notify the IRB and the FDA accordingly. As part of the pause review, the reviewers will also advise on whether the study needs to be paused again for any subsequent AEs of the same type. The pause criterion for SAEs will continue to apply.

The administration of study injections and new enrollments would resume only if review of the events that caused the pause resulted in a recommendation to permit further enrollments and study injections. Safety data reports and changes in study status will be submitted to relevant regulatory authorities in accordance with Section 5.0 and institutional policy.

5. SAFETY AND ADVERSE EVENT REPORTING

5.1 Adverse Events

Adverse Event (AE) - Any untoward medical occurrence in a human subject, including any abnormal sign (for example, abnormal physical exam or laboratory finding), symptom, or disease, temporally associated with the subject's participation in research, whether or not considered related to the subject's participation in the research. In the context of FDA-required reporting, an AE means any untoward medical occurrence associated with the use of a drug in humans, whether or not considered drug related. All AEs, including laboratory abnormalities, will be followed to resolution or stabilization.

Each AE will be graded according to the Toxicity Grading Scale for Healthy Adult and Adolescent Volunteers Enrolled in Preventive Vaccine Clinical Trials, Food and Drug Administration Guidance – September 2007, APPENDIX II. The following guidelines will be used to determine whether or not an AE is recorded in the study database:

1. Solicited AEs (i.e., reactogenicity parameters as defined in Section 4.4.10) will be recorded without attribution assessments by the subject on paper or an electronic diary card for 7 days after each injection. If the paper diary card is completed by a subject, data will be transcribed by a clinician into the study database. Clinicians will follow and collect resolution information for any reactogenicity symptoms that are not resolved within 7 days.
2. Unsolicited AEs will be recorded with assessments of relatedness to study product, severity and seriousness (per Section 5) by the investigator in the study database from receipt of the study injection through completion of the 4-week post-product administration visit. After that through the last study visit, only SAEs (Section 5.2), adverse events of special interest (AESI, Section 5.4), AEs leading to withdrawal, and new onset chronic medical conditions will be recorded.
3. Medically attended AEs (MAAEs, Section 5.5) will be collected through 6 months after the last vaccination.

5.2 Serious Adverse Events

The term "Serious Adverse Event" (SAE) is defined in 21 CFR 312.32 as follows: "An adverse event or suspected adverse reaction is considered serious if, in the view of either the investigator or the sponsor, it results in any of the following outcomes: Death, a life-threatening adverse event, inpatient hospitalization or prolongation of existing hospitalization, a persistent or significant incapacity or substantial disruption of the ability to conduct normal life functions, or a congenital anomaly/birth defect. Important medical events that may not result in death, be life-threatening, or require hospitalization may be considered serious when, based upon appropriate medical judgment, they may jeopardize the patient or subject and may require medical or surgical intervention to prevent one of the outcomes listed in this definition. Examples of such medical events include allergic bronchospasm requiring intensive treatment in an emergency room or at home, blood dyscrasias or convulsions that do not result in inpatient hospitalization, or the development of drug dependency or drug abuse."

"Life threatening" refers to an AE or suspected adverse reaction that represents an immediate risk of death to the subject. It does not include an AE or suspected adverse reaction that, had it occurred in a more severe form, might have caused death. Similarly, a hospital admission for an elective procedure is not considered a SAE.

5.3 Adverse Event Reporting to the IND Sponsor

AEs that meet SAE criteria must be reported and submitted by the clinical site on an expedited basis to the IND Sponsor, DMID, according to sponsor guidelines as follows:

- Results in death,
- Is life threatening (places the subject at immediate risk of death from the event as it occurred),
- Results in inpatient hospitalization or prolongation of existing hospitalization,
- Results in a persistent or significant disability/incapacity,
- Results in a congenital anomaly/birth defect in the offspring of a study subject, OR
- Based upon appropriate medical judgment, may jeopardize the subject's health and may require medical or surgical intervention to prevent one of the other outcomes listed in this definition (examples of such events include allergic bronchospasm requiring intensive treatment in the emergency room or at home, blood dyscrasias or convulsions that do not result in inpatient hospitalization, or the development of drug dependency or drug abuse).
- In addition, any event, regardless of severity, which in the judgment of an investigator represents a SAE, may be reported on an expedited basis.

An investigator will communicate an initial SAE report within 24 hours of site awareness of occurrence to the IND Sponsor by data entry into the database, which triggers an alert to the IND Sponsor Medical Officer (DMID Medical Monitor) and DMID Pharmacovigilance (Section 5.6). Within 3 working days, a written summary by the investigator should be submitted to the IND Sponsor.

In order for the IND Sponsor to comply with regulations mandating sponsor notification of specified SAEs to the FDA within 7 and/or 15 calendar days, the investigator must submit additional information as soon as it is available.

5.4 Adverse Events of Special Interest (AESI)

An AESI is an AE (serious or nonserious) of scientific medical concern specific to the Sponsor's product or program, for which ongoing monitoring and immediate notification by the investigator to the Sponsor is required. Such events may require further investigation to characterize and understand them. A list of additional AESI that should be prospectively collected is included in APPENDIX III. AESIs are collected through the entire study period and recorded in the database. AESIs that are also SAEs must be reported to the Sponsor or designee within 24 hours of site awareness of the event via the electronic data capture (EDC) system. The investigator will continue to submit any updated information on AESIs that are SAEs to the Sponsor as it becomes available.

5.4.1 Anaphylaxis

All suspected cases of anaphylaxis should be recorded as AESIs and reported as SAEs. For reporting purposes, a participant who displays signs/symptoms consistent with anaphylaxis as shown below should be reported as a potential case of anaphylaxis. This is provided as a general guidance for investigators and is based on the Brighton Collaboration case definition [46].

Anaphylaxis is an acute hypersensitivity reaction with multi organ system involvement that can present as, or rapidly progress to, a severe life-threatening reaction. It may occur following exposure to allergens from a variety of sources. Anaphylaxis is a clinical syndrome characterized by:

- Sudden onset AND
- Rapid progression of signs and symptoms AND
- Involving 2 or more organ systems, as follows:
    - Skin/mucosal: urticaria (hives), generalized erythema, angioedema, generalized pruritus with skin rash, generalized prickle sensation, red and itchy eyes
    - Cardiovascular: measured hypotension, clinical diagnosis of uncompensated shock, loss of consciousness or decreased level of consciousness, evidence of reduced peripheral circulation
    - Respiratory: bilateral wheeze (bronchospasm), difficulty breathing, stridor, upper airway swelling (lip, tongue, throat, uvula, or larynx), respiratory distress, persistent dry cough, hoarse voice, sensation of throat closure, sneezing, rhinorrhea
    - Gastrointestinal: diarrhea, abdominal pain, nausea, vomiting

5.4.2 Myocarditis and Pericarditis

A case of suspected, probable, or confirmed myocarditis, pericarditis, or myopericarditis should be reported as an AESI, even if it does not meet criteria per the CDC case definition (see APPENDIX III) [47, 48]. The event should also be reported as an SAE if it meets seriousness criteria (Section 5.2).

5.5 Medically Attended Adverse Events (MAAEs)

MAAEs are defined as adverse events leading to hospitalization, an emergency room visit or an otherwise unscheduled visit to or from medical personnel, for any reason. The MAAEs will be collected and recorded in the database in a period from the first vaccination to 6 months after the last vaccination.

5.6 Reporting of Serious Adverse Events to DMID Pharmacovigilance

SAEs will be followed until resolution even if this extends beyond the study-reporting period. Resolution of an AE is defined as the return to pretreatment status or stabilization of the condition with the expectation that it will remain chronic.

Any AE that meets a protocol-defined serious criterion must be submitted immediately (within 24 hours of site awareness) on an SAE form to the DMID Pharmacovigilance Group, at the following address:

DMID Pharmacovigilance Group

Clinical Research Operations and Management Support (CROMS)

6500 Rock Spring Dr. Suite 650

Bethesda, MD 20817, USA

SAE Hot Line: 1-800-537-9979 (US) or 1-301-897-1709 (outside US)

SAE FAX Number: 1-800-275-7619 (US) or 1-301-897-1710 (outside US)

SAE Email Address: PVG@dmidcroms.com

Other supporting documentation of the event may be requested by the DMID Pharmacovigilance Group and should be provided as soon as possible.

In addition to the VRC notification, the DMID Medical Monitor and DMID Clinical Project Manager will be informed of the SAE by the DMID Pharmacovigilance Group. The DMID Medical Monitor will review and assess the SAE for regulatory reporting and potential impact on study subject safety and protocol conduct.

At any time after completion of the study, if the site principal investigator or appropriate sub-investigator becomes aware of an SAE that is suspected to be related to study product, the site principal investigator or appropriate sub-investigator will report the event to the DMID Pharmacovigilance Group.

5.7 Reporting of Pregnancy

Pregnancy is not an adverse event, but pregnancy, if occurred, will be recorded in the study database, and subjects will be followed for clinical safety and to collect the pregnancy outcome as described in Section 4.4.7. Pregnancy itself is not regarded as an SAE. However, congenital abnormalities or birth defects and spontaneous miscarriages that meet serious criteria (Section 5.2) should be reported as SAEs.

Pregnancy, if occurred, will be reported to the Sponsor no later than 24 hours after investigator awareness. The Investigator will notify the Sponsor no later than 24 hours of when the outcome of the pregnancy becomes known.

Pregnancy will be reported to regulatory agencies.

5.8 IND Sponsor Reporting to the FDA

The IND Sponsor is responsible for making the determination of which SAEs are “serious and unexpected suspected adverse reactions” (SUSARs) as defined in 21 CFR 312.32. The following definitions apply:

- *Suspected Adverse Reaction* means any AE for which there is a reasonable possibility that the drug caused the AE.

- *Unexpected Adverse Event* means an AE that is not listed (refer to Risks in Section 8.5) at the specificity or severity that has been observed.

All SUSARs (as determined by the IND Sponsor) will be reported to the FDA as IND Safety Reports per 21 CFR 312.32 as soon as possible but not exceeding 7 calendar days for unexpected fatal or life-threatening events, and not exceeding 15 calendar days for other qualifying events. IND Safety Reports will also be provided to the IRB.

The IND Sponsor will also submit an IND Annual Report of the progress of the investigation to the FDA as defined in 21 CFR 312.33.

5.9 Reporting to the Institutional Review Board

The following information is consistent with NIH IRB Policy 801: Reporting Research Events.

Reportable Event - An event that occurs during the course of human subject research that requires notification to the IRB.

For the purposes of this policy, reportable events include the following:

- Unanticipated Problems (UPs) involving risks to subjects or others
- Non-compliance (including major protocol deviations and noncompliance that is not related to a protocol deviation)
- Deaths related or possibly related to research activities
- New information that might affect the willingness of subjects to enroll or continue participation in the study
- Suspension or termination of research activities

5.9.1 Unanticipated Problem

An Unanticipated Problem (UP) is defined as any incident, experience, or outcome that meets all the following criteria:

- Unexpected (in terms of nature, severity, or frequency) given (a) the research procedures that are described in the protocol-related documents, such as the IRB approved research protocol and informed consent document; and (b) the characteristics of the subject population being studied; and
- Related or possibly related to participation in the research (possibly related means there is a reasonable possibility that the incident, experience, or outcome may have been caused by the procedures involved in the research); and
- Suggests that the research places subjects, or others (which may include research staff, family members or other individuals not directly participating in the research) at a greater risk of harm (including physical, psychological, economic, or social harm) related to the research than was previously known or expected.

A UP must be reported within 7 calendar days of an investigator becoming aware of the actual or suspected UP.

5.9.2 Non-Compliance

Non-compliance is the failure of investigator(s) to follow the applicable laws, regulations, or institutional policies governing the protection of human subjects in research, or the requirements or determinations of the IRB, whether intentional or not.

Non-compliance may be unintentional (e.g. due to lack of understanding, knowledge, or commitment), or intentional (e.g. due to deliberate choice to ignore or compromise the requirements of any applicable regulation, organizational policy, or determination of the IRB).

Non-compliance is further characterized as serious or continuing as follows:

- Serious non-compliance – Non-compliance, whether intentional or not, that results in harm or otherwise materially compromises the rights, welfare and/or safety of the subject. Non-compliance that materially effects the scientific integrity or validity of the research may be considered serious non-compliance, even if it does not result in direct harm to research subjects.
- Continuing non-compliance – A pattern of recurring non-compliance that either has resulted, or, if continued, may result in harm to subjects or otherwise materially compromise the rights, welfare and/or safety of subjects, affect the scientific integrity of the study or validity of the results. The pattern may comprise repetition of the same non-compliant action(s), or different noncompliant events.

Any actual or suspected non-compliance by any investigator or entity associated with the protocol must be reported to the IRB by the PI/designee within 7 calendar days of any investigator or individual associated with the protocol first becoming aware.

5.9.3 Protocol Deviation

A Protocol Deviation (PD) is defined as any change, divergence, or departure from the IRB-approved research protocol and are further characterized as major and minor as follows:

- Major Deviations – Deviations from the IRB approved protocol that have, or may have the potential to, negatively impact, the rights, welfare or safety of the subject, or to substantially negatively impact the scientific integrity or validity of the study.
- Minor Deviations – Deviations that do not have the potential to negatively impact the rights, safety, or welfare of subjects or others, or the scientific integrity or validity of the study.

For the reporting purposes, failure of subjects to comply with the research protocol does not represent non-compliance unless that failure is due to an action or omission of a member of the research team, for example, the failure to give adequate instruction to the subject.

A major deviation must be reported within 7 calendar days of an investigator becoming aware of an actual or suspected deviation. Although PDs are also non-compliance, these should only be reported once as deviations. Major deviations resulting in death must be reported within 24 hours of the occurrence of the event or of any member of the study team becoming aware of the death.

Researchers are responsible for monitoring their studies throughout the year for adherence to the IRB approved protocol. The purpose of this monitoring is to identify major deviations and to

look for trends in minor deviations that may indicate a systemic issue in how the study is being conducted that could potentially negatively impact the rights, safety, or welfare of participants or the study's ability to produce scientifically valid results. A series of minor deviations pointing toward a more global issue that could affect the rights, safety or welfare of the participant or affect the validity of the study should be reported as a major deviation. In all other instances, a summary of minor deviations should be provided to the IRB at the time of continuing review.

5.9.4 Death

Any death of a research subject that is possibly, probably or definitely related to the research must be reported within 24 hours of an investigator becoming aware of the death.

5.9.5 New Information

New information that might affect the willingness of a subject to enroll or remain in the study should be reported within 7 calendar days of an investigator first becoming aware.

5.9.6 Suspension or Termination of Research Activities

Any suspension or termination of research activities, including holds on new enrollment, placed upon the research by the study sponsor, NIH or IC leadership, or any regulatory agency must be reported within 7 calendar days of an investigator becoming aware.

5.9.7 Expedited Reporting to the IRB

Death related to research must be reported within 24 hours.

The following will be reported within 7 calendar days of investigator awareness:

- Actual or suspected UPs,
- Actual or suspected non-compliance,
- Actual or suspected Major PDs,
- SAEs that are actual or suspected UPs,
- New information that might affect the willingness of a subject to enroll or remain in the study,
- Suspension or termination of research activities, including holds on new enrollment, placed upon the research by the study sponsor, NIH or IC leadership, or any regulatory agency.

5.9.8 Annual Reporting to the IRB

The following will be reported to the IRB in summary at the time of Continuing Review:

- Summary of UPs and non-compliance,
- AEs, including SAEs, that are not UPs, as a narrative summary statement indicating whether these events were within the expected range,
- Minor PDs (aggregate summary),
- Any trends or events which in the opinion of the investigator should be reported.

6. STATISTICAL CONSIDERATIONS

6.1 Overview

This is a Phase I, dose escalation, open-label clinical trial to evaluate dose, safety, tolerability and immunogenicity of a NiV mRNA vaccine, mRNA-1215.

6.2 Sample Size and Accrual

Recruitment will target 40 healthy adult participants between 18 to 60 years of age. Up to 50 participants may be enrolled if deemed necessary for safety or immunogenicity evaluations. Each study group will recruit 10 healthy subjects.

6.3 Endpoints

6.3.1 Primary Endpoints: Safety

Assessment of product safety will include clinical observation and monitoring of hematological and chemical parameters. APPENDIX I provides details and specified time points for medical assessments. The following endpoints will be assessed for all study groups:

- Frequency and severity of solicited local reactogenicity symptoms reported for 7 days after each product administration
- Frequency and severity of solicited systemic reactogenicity symptoms reported for 7 days after each product administration
- Frequency and grade of any unsolicited AEs, including abnormal safety laboratory measures, during the 28-day follow-up period post each vaccination
- Frequency of serious adverse events (SAEs), AESIs, adverse events leading to withdrawal, and new chronic medical conditions through the last study visit
- Frequency of MAAEs through 6 months after last vaccination

6.3.2 Secondary Endpoints: Immunogenicity

Secondary objectives will evaluate binding antibody responses to the mRNA-1215 vaccine (pre-F and G proteins) at doses of 10 mcg, 25 mcg, 50 mcg and 100 mcg at 2 weeks after last product administration as follows:

- Geometric Mean Titer (GMT) with 95% CI for the NiV-specific antibodies will be reported for study groups as appropriate.

6.3.3 Exploratory Endpoints: Immunogenicity

Exploratory endpoints may evaluate the specificity and functionality of mRNA-1215 vaccine-induced antibodies and the immune response at various timepoints throughout the study. Additionally, exploratory endpoints may also evaluate the frequency, magnitude, phenotype, and specificity of B-cell, T-cell, and antibody responses at various time points throughout the study.

6.3.4 Sample Size Consideration for Safety

For each group (dose escalation) with sample size n=10, there is over 90% chance to observe at least 1 SAE if the true rate is at least 0.21 and over 90% chance to observe no SAE if the true rate is no more than 0.01. Probabilities of observing 0 or more than 1 SAE within a group are presented in **Table 2** for a range of possible true event rates.

Table 2: Probability of Observing Events under Different Scenarios within a Group (n=10)

True Event Rate	Groups 1-4, n=10	
	Pr(0)	Pr(>1)
0.005	0.951	0.001
0.01	0.904	0.004
0.02	0.817	0.016
0.03	0.737	0.035
0.04	0.665	0.058
0.05	0.599	0.086
0.1	0.349	0.264
0.15	0.197	0.456
0.2	0.107	0.624
0.3	0.028	0.851
0.4	0.006	0.954
0.5	0.001	0.989

Table 3 gives the upper and lower bounds for 95% exact binomial confidence intervals of the true SAE rate at possible numbers of events within a group. Within a group of size n=10, if none experience an SAE, the 95% exact confidence interval has upper bound 0.308. Within a group of size n=10, if 2 enrollees experience an SAE, the exact 95% confidence interval has lower bound 0.025 and upper bound 0.556.

Table 3: 95% Confidence Intervals for True Rate under Possible Observed Number of Events

Observed Number of Events	95% Confidence Interval (n=10)	
	Lower Bound	Upper Bound
0	0.000	0.308
1	0.003	0.445
2	0.025	0.556
3	0.067	0.652
4	0.122	0.738
5	0.187	0.813
6	0.262	0.878
7	0.348	0.933
8	0.444	0.975
9	0.555	0.997
10	0.692	1.000

6.3.5 Sample Size Consideration for Immunogenicity

Table 2 also applies to the sample size consideration for immunogenicity. Within a group of size n=10, if there are two positive responders with respect to an immunogenicity endpoint, the 95% confidence interval of the positive response rate has the lower bound of 0.025 and the upper bound of 0.556.

6.3.6 Sample Size Consideration for Comparison

For the comparison of positive response rate between three arms each of size n=10, we present in Table 3 the minimum event rate that can be detected with 80% or 90% power in the high-responding arm over a range of possible event rates in the low-responding arm. If the positive response rate is 0.1 in the low-responding arm, this trial has 80% (or 90%) power to detect the between-arm difference if the positive response rate in the other arm is no less than 0.68 (or 0.75). This calculation was performed based on Fisher's exact test.

For the comparison of the magnitude of immune response between two arms each of size n=10, the trial has 80% (or 90%) power to detect the between-arm difference if the effect size is no less than 1.20 (or 1.38); that is, the mean difference is no less than 1.20 (or 1.38) times the standard deviation of the immune response. This power calculation is based on two-sample t-test, and a log-transformation of the immune response may be needed if the data is close to being log-normally distributed.

Table 4: Minimum Event Rate That Can Be Detected With 80% Or 90% Power with Sample Size n=10 per Arm

Rate in one arm	Minimum rate in the other arm (80% power)	Minimum rate in the other arm (90% power)
0.1	0.78	0.84
0.2	0.88	0.93
0.3	0.95	0.985
0.4	0.995	-

Note: If the rate is 0.4, with a rate of 1 in the other arm the highest power that can be achieved is 0.84.

6.4 Statistical Analysis

All statistical analyses will be performed using Statistical Analysis System (SAS) (SAS Institute, Cary, NC), R, or S-Plus statistical software. No formal multiple comparison adjustments will be employed for safety endpoints or secondary endpoints.

6.4.1 Analysis Variables

The analysis variables consist of baseline variables and safety variables for primary and secondary objective analyses.

6.4.2 Baseline Demographics

Baseline characteristics including demographics and laboratory measurements will be summarized using descriptive statistics.

6.4.3 Safety Analysis

6.4.3.1 Solicited Reactogenicity

The number and percentage of participants experiencing each type of reactogenicity sign or symptom will be tabulated by severity. For a given sign or symptom, each participant's reactogenicity will be counted once under the maximum severity for all assessments.

6.4.3.2 Adverse Events

AEs are coded into Medical Dictionary for Regulatory Activities (MedDRA) preferred terms. The number and percentages of participants experiencing each specific AE will be tabulated by severity and relationship to treatment. For the calculations in these tables, each participant's AE will be counted once under the maximum severity or strongest recorded causal relationship to treatment.

A complete listing of AEs for each participant will provide details including severity, relationship to treatment, onset, duration and outcome.

6.4.3.3 Local Laboratory Values

Boxplots, violin plots, or beeswarm plots of local laboratory values will be generated for baseline values and for values measured during the course of the study. Each plot will show the 1st quartile, the median, and the 3rd quartile. Outliers, or values outside the boxplot, will also be plotted. If appropriate, horizontal lines representing boundaries for abnormal values will be plotted.

6.4.4 Immunogenicity Analysis

The statistical analysis for immunogenicity will employ the intent-to-treat principle whereby all data from enrolled subjects will be analyzed according to the group assignment. If needed, a per-protocol analysis will be performed as secondary analysis where subjects will be analyzed according to their actual vaccination scheme if it is different from the assigned or up to the last visit in the study if there are early dropouts.

If assay data are qualitative or dichotomous (i.e., positive or negative) then analyses will be performed by tabulating the frequency of positive response for each assay at each time point that an assessment is performed. Binomial response rates will be presented with their corresponding exact 95% confidence interval estimates.

Some immunologic assays have underlying continuous or count-type readout that is often dichotomized into responder/non-responder categories. For these assays, graphical and tabular summaries of the underlying distributions will be made. These summaries may be performed on transformed data (e.g., log transformation) for ease of interpretation.

6.4.5 Missing Data

Missing responses will be assumed to be missing completely at random. Analyses will include all samples available at each study time point. Based on experience from previous trials, we expect missing data to be rare. Regardless, in the event of missing data, we will report the

occurrence and extent of missingness. We will also provide plausible explanations for the missingness mechanism, should such information be available.

6.4.6 Interim Analyses

Safety Reviews: The PSRT will review safety data routinely throughout the study. The study will utilize both electronic database features and reviews by designated safety review personnel to identify in a timely manner if any of the safety pause rules of the study are met.

Immunogenicity Review: The mRNA-1215 dose of 10 mcg was chosen for Group 4 following an interim analysis of safety and immunogenicity data from enrollments in Groups 1-3 (Section 4.6). Other interim analyses of immunogenicity may be performed prior to completion of safety follow-up visits or collection of data for secondary and exploratory immunogenicity endpoints.

7. PHARMACY AND VACCINE ADMINISTRATION PROCEDURES

The study groups and study agent dosing schedule are shown in Table 1 in Section 4.1.

7.1 mRNA-1215 Vaccine, Pharmaceutical Formulation

mRNA-1215 vaccine (also referred to as mRNA-1215 Injection) consists of mRNA encapsulated in a LNP and is administered by intramuscular (IM) injection. The mRNA Drug Substance is encapsulated by LNPs composed of four lipids: one of the lipids is the proprietary ionizable lipid, SM-102, and three are commercially available lipids, cholesterol, DSPC, and PEG2000 DMG. mRNA-1215 is sterile filtered and filled into glass vials at a total mRNA concentration of 0.40 mg/mL and a volume of 0.8 mL.

The vaccine manufacturer, ModernaTX, Inc, will deliver the vaccine vials to the DMID Clinical Materials Repository at Thermo Fisher Scientific, Germantown, MD, from where the vaccine will be distributed to the site.

7.2 Vaccine Storage

mRNA-1215 is stored at -70°C (-90°C to -60°C). Stability protocols for mRNA-1215 will include at least 24-months duration at the intended storage temperature (-90°C to -60°C).

The prepared doses are stable for clinical in-use for up to 8 hours at room temperature.

7.2.1 Handling Information

mRNA-1215 must be stored in a secure area with limited access (pharmacy staff only). The freezer should have an automated temperature recording and alert system. There must be an available back-up freezer. The freezers must be connected to a back-up generator, or alternate plan in the event of a power failure. The pharmacy must have in place a 24-hour alert system that allows for rapid response in case of freezer malfunctioning.

Prepared doses should remain at ambient temperatures (20ºC to 25ºC) and should not be shaken or allowed to roll during transport. Avoid exposing to direct sunlight while the syringe is in route to the injection administration location. If the prepared doses require transportation to a separate facility, ensure there are appropriate transport procedures in place. Under all circumstances, the time for preparation, transportation and administration of mRNA-1215 must occur within <u>maximum 9 (nine) hours</u> from the time the vials are removed from the freezer, including about 1 hour for a dose preparation and 8 hours stability of the prepared doses.

A minimum of 20 minutes and a maximum of 30 minutes are allowed for equilibration of vial to controlled room temperature (20°C to 25°C) after removal from freezer. A maximum of 30 minutes is allowed between completion of equilibration and completion of dosing syringes fill. However, all dosing syringes must be filled in quick succession once the vial is breached.

7.2.2 Temperature Excursions

If deviations in storage temperature occur from the normal allowance for the pharmacy freezer, the site pharmacist or designee must report the storage temperature excursion promptly to the PI

and IND Sponsor. The excursion must be evaluated and investigated, and action must be taken to restore and maintain the desired temperature limits. Pending the outcome of the investigation, the IND Sponsor will notify the pharmacist if continued clinical use of the product is acceptable.

In the case of storage or shipping/handling temperature excursions outside of the normal allowance for the storage device, the following procedure is to be followed:

1. Quarantine the affected product in a separate area. If the excursion results in thawed material for the investigational products, it must not be refrozen. Thawed vials must be quarantined at 5°C ± 3°C. Frozen vials must be quarantined at the labeled storage temperature of -70°C (-90°C to -60°C).
2. Report the excursion to the IND sponsor's authorized representative (SAR) or designee, any other parties required by site procedures, and via email to DMIDProductSupportTeam@niaid.nih.gov. Do not use the product until the IND Sponsor's Authorized Representative (SAR) or designee informs the site pharmacist whether continued clinical use of the product is acceptable.
3. After receipt and evaluation of the reported information, the Sponsor or manufacturer's designee will notify the site pharmacist whether continued clinical use of the product is acceptable.
4. The DMID process for temperature excursions is procedural and will be provided to the site.

7.3 Preparation of mRNA-1215 for Administration

Pharmacists must follow the detailed instructions in the mRNA-1215 vaccine Preparation and Administration Worksheets. There are 4 worksheets in total, one for each dose level. The mRNA-1215 vaccine Dose Preparation and Administration Worksheets are in the Study Specific Procedures (SSP).

Below are general guidelines for the product preparation.

- One vial of vaccine is required to prepare each dose.
- Pharmacists must follow appropriate aseptic technique and sterile preparation procedures/guidance as outlined in USP <797> [medium risk], utilizing a pharmacy biosafety cabinet/isolator or better. Local regulations and site institutional policies and procedures for use of personal protective equipment, such as gloves, gowns, masks, and safety glasses, must be followed. Pharmacists should follow the requirements of their country, their institution, and their pharmacy regulatory authority regarding these procedures.

Any unused portion of study product will not be used for another participant. Empty vials, unused portion of entered vials, or unused prepared study product should be discarded in a biohazard container and disposed of in accordance with institutional or pharmacy policy.

7.3.1 Dose Preparation

The mRNA-1215 doses for administration in study groups will be prepared as described in Table 5 below.

Table 5: mRNA-1215 Injection Dose Preparation Schema

Dose	Diluent (0.9% Sodium Chloride) volume (mL)	mRNA 1215 volume (mL)	Total volume in mixing vial (mL)	Injection Dose Volume (mL)	Dosing Syringe Expiration (hours)
25 mcg	1.75	0.25	2	0.5	8
50 mcg	1.50	0.50	2	0.5	8
100 mcg	0.50	0.50	1	0.5	8
10 mcg	3.8	0.2	4	0.5	8

Detailed instructions for dose preparations are provided in the Pharmacy Manual.

7.4 Administration of mRNA-1215 Injection

Clinician instructions on how to select an arm and administer an IM injection are in Section 4.4.8. Subjects will receive the vaccine by needle and syringe injection in either deltoid. Clinicians will choose the appropriate needle size for each subject. Product labeling verification and IM injection procedures will be performed consistent with institutional policies and standard procedures.

7.5 Study Product Accountability

7.5.1 Documentation

The study pharmacist or designee will be responsible for maintaining an accurate record of the codes, inventory, and an accountability record of the investigational study products supplies for this study. Electronic documentation as well as paper copies will be used.

7.5.2 Disposition

Empty vials and the unused portion of a vial will be discarded in a biohazard containment bag and incinerated or autoclaved in accordance with the institutional or pharmacy policy. Partially used vials will not be administered to other subjects or used for in vitro experimental studies.

Any unopened vials that remain at the end of the study may be returned to DMID or discarded at the discretion of the sponsor in accordance with policies that apply to investigational agents. Vials will be disposed of in accordance with institutional or pharmacy policy.

8. HUMAN SUBJECT PROTECTIONS AND ETHICAL OBLIGATIONS

This research study will be conducted in compliance with the protocol, International Council for Harmonisation Good Clinical Practices (ICH-GCP) guidance, and all applicable regulatory requirements.

8.1 Institutional Review Board

A copy of the protocol, ICF, other written subject-facing information, and any advertising material will be submitted to the IRB for written approval prior to use.

The PI must submit and, where necessary, obtain approval from the IRB for all subsequent protocol amendments and changes to the ICF. The PI will notify the IRB of research events that occur on study as described in Section 5.8.

The investigator will be responsible for obtaining IRB approval of the annual Continuing Review throughout the duration of the study.

8.2 Informed Consent

The PI or designee is responsible for obtaining written informed consent from the subject after adequate explanation of the aims, methods, anticipated risks and benefits of the study and before any protocol-specific procedures or study product is administered. The AoU must be completed before the study ICF is signed.

The study informed consent form (ICF) describes the investigational product to be used and all aspects involved in protocol participation. It is provided to potential participants for their review in advance, typically as a hard copy during screening as described in Section 4.4.4. Volunteers will have ample time to ask questions and discuss this study with NIH staff, and with their family, friends, and personal health care providers prior to signing the informed consent.

Informed consent process is to be conducted in person, with only relevant parties present, to protect the privacy of the subject. The study clinicians that are listed as Associate Investigators on the study are authorized to obtain consent and to respond to volunteers' questions as needed. Steps to reduce coercion or undue influence for volunteers who are NIH employees are described in the protocol Section 4.3.4.

The acquisition of informed consent will be documented in the subject's medical records, as required by 21 CFR 312.62, and the ICF will be signed and personally dated by the subject and the person who conducted the informed consent discussion. A copy of the signed ICF will be given to the subject and the original will be scanned into the electronic medical record.

8.3 Study Discontinuation and Closure

This study may be temporarily suspended or prematurely terminated if there is sufficient reasonable cause. Written notification, documenting the reason for study suspension or termination, will be provided by the suspending or terminating party to study participants, the investigators, the Investigational New Drug (IND) and regulatory authorities as appropriate. If

the study is prematurely terminated or suspended, the PI will promptly inform study participants, the IRB, and Sponsor and will provide the reason(s) for the termination or suspension. Study participants will be contacted, as applicable, and be informed of changes to study visit schedule.

Circumstances that may warrant termination or suspension include, but are not limited to:

- Determination of unexpected, significant, or unacceptable risk to participants
- Demonstration of efficacy that would warrant stopping
- Insufficient compliance to protocol requirements
- Data that are not sufficiently complete and/or evaluable
- Determination that the primary endpoint has been met
- Determination of futility

The study may resume once concerns about safety, protocol compliance, and data quality are addressed, and satisfy the Sponsor, IRB, Office for Human Research Protections (OHRP), and/or FDA.

8.4 Confidentiality and Privacy

Participant confidentiality and privacy is strictly held in trust by the participating investigators, their staff, and the Sponsor(s) and their representatives. This confidentiality is extended to cover testing of biological samples and genetic tests in addition to the clinical information relating to participants. Therefore, the study protocol, documentation, data, and all other information generated will be held in strict confidence. No information concerning the study, or the data will be released to any unauthorized third party without prior written approval of the IND sponsor.

All research activities will be conducted in as private a setting as possible.

The study monitor, other authorized representatives of the Sponsor (DMID), representatives of the VRC, representatives of the IRB, and/or regulatory agencies may inspect all documents and records required to be maintained by the investigator, including but not limited to, medical records (office, clinic, or hospital) and pharmacy records for the participants in this study. The clinical study site will permit access to such records.

The study participant's contact information will be securely stored at the clinical site for internal use during the study. At the end of the study, all records will continue to be kept in a secure location for as long a period as dictated by the reviewing IRB, Institutional policies, or Sponsor requirements.

Study participant research data, which is for purposes of statistical analysis and scientific reporting, will be transmitted to and stored by The Emmes Company, LLC (Rockville, MD), the Data Coordinating Center. This will not include the participant's contact or identifying information. Rather, individual participants and their research data will be identified by a unique study identification number. The study data entry and study management systems used by the clinical site and by Emmes research staff will be secured and password protected. At the end of the study, all study databases will be de-identified and archived.

To further protect the privacy of study participants, a Certificate of Confidentiality has been issued by the National Institutes of Health (NIH). This certificate protects identifiable research information from forced disclosure. It allows the investigator and others who have access to research records to refuse to disclose identifying information on research participation in any civil, criminal, administrative, legislative, or other proceeding, whether at the federal, state, or local level. By protecting researchers and institutions from being compelled to disclose information that would identify research participants, Certificates of Confidentiality help achieve the research objectives and promote participation in studies by helping assure confidentiality and privacy to participants.

8.5 Risks and Benefits Assessment

8.5.1 Risks of mRNA-1215

This is the first study in humans of the investigational vaccine, mRNA-1215, and therefore risks are unknown at the time of study start. The risks listed below are those associated with the administration of vaccines in general, including other mRNA-based vaccines.

Subjects may exhibit general signs and symptoms associated with vaccine administration, including fever, chills, rash, nausea/vomiting, headache, dizziness, joint pain, and fatigue. Potential side effects of IM vaccine injection include pain/tenderness, stinging, pruritis, arm discomfort, redness of the skin or mild bruising at vaccine injection site. Axillary lymphadenopathy on the same side as vaccination has also occurred with other mRNA vaccines. These side effects will be monitored, but are generally short term, mild to moderate severity and usually do not require treatment.

There is a very small chance of allergic reactions, including anaphylaxis, as has been observed with mRNA platforms and other vaccines. There is also a rare possibility of developing chronic urticaria.

There have been very rare reports of myocarditis and pericarditis occurring after vaccination with COVID-19 mRNA vaccines. The majority of the cases have been reported in males and females shortly after the second dose of the vaccine. The risk is highest in males under the age of 40 years, specifically males between 12 through 17 years of age. Symptoms of myocarditis or pericarditis include chest pain, shortness of breath, or feelings of having a fast-beating, fluttering, or pounding heart, with onset of symptoms most commonly reported within a few days following vaccination. Study participants should seek medical attention and also notify study site staff if any of these symptoms occur following vaccination. While some cases required intensive care support, available data from short-term follow up suggest that most cases had resolution of symptoms with conservative management. Information is not yet available about potential long-term sequelae. It is not known whether the risk of myocarditis or pericarditis is increased following additional doses of the vaccine. Investigators and study participants should be alert to the signs and symptoms of myocarditis and pericarditis [48].

There may be other unknown side effects.

8.5.2 Risks of Specimen Collection

- *Blood drawing*: The risks of blood sample collection are minimal and consist of mild discomfort at the sample collection site. The procedure may cause pain, bruising, lightheadedness or fainting, and, rarely, infection at the site where the blood is taken.
- *Apheresis*: The procedure may cause pain, bruising, and discomfort in the arms where the needles are placed. It may also cause chills, nausea, heartburn, mild muscle cramps and tingling sensation around the mouth or in the fingers, however this can usually be relieved by slowing or temporarily interrupting the apheresis procedure or taking a calcium containing antacid, such as Tums®. Other possible side effects are anxiety, vomiting and lightheadedness. Temporary lowering of the blood pressure may develop. There is the rare possibility of infection, fainting or seizure. Very rarely a nerve problem at the needle placement site may occur. Also, very rarely, a machine malfunction may occur, resulting in the loss of about one unit of blood. There may be additional risks of apheresis that are unknown at this time.

8.5.3 Risks of mRNA-1215 for the Fetus or Nursing Infant

We do not know the possible effects of the study vaccine on the fetus or nursing infant. Women of reproductive potential will be required to agree to use an effective method of birth control beginning 21 days prior to enrollment and continuing through end of study.

Because this is a research study, women of reproductive potential will be tested for pregnancy prior to administration of study injection and asked to notify the site immediately upon learning of a pregnancy during this study. In the case of pregnancy, subjects will continue to be followed for safety. Research sample collections, study procedures and additional product administrations will be discontinued for pregnant women. The subject will be contacted to ask about the outcome of a pregnancy that begins during the study.

8.5.4 Risks of New Diagnoses

It is possible that the standard medical tests performed as part of this research protocol will result in new diagnoses. Depending upon the medical findings and consequences of being provided with the new medical information about health status, the study subject may view this aspect of study participation as either a risk or a benefit. Any such information will be shared and discussed with the subject and, if requested by the subject, will be forwarded to the subject's primary health care provider for further workup and management.

8.5.5 Potential Benefits

Study subjects will not receive direct health benefit from study participation. This protocol is not designed to provide treatment for any condition. Others may benefit from knowledge gained in this study that may aid in the development of a Nipah Virus vaccine. The investigational vaccine is not expected to provide protection from Nipah Virus infection.

8.5.6 Assessment of Potential Risks and Benefits

This healthy volunteer trial to evaluate the safety and immunogenicity of mRNA-1215 was reviewed using the VRC Risk Management Plan. Potential risks, acceptance of risks, and mitigation strategies are available in the VRC 322/DMID 21-0016 Risk Register.

8.6 Plan for Use and Storage of Biological Samples

The plan for use and storage of biological samples from this protocol is as outlined in the following sections.

8.6.1 Use of Samples, Specimens and Data

Samples, specimens and data collected under this protocol may be used to conduct protocol-related safety, and immunogenicity evaluations, exploratory laboratory evaluations related to NiV, exploratory laboratory evaluations related to mAb, vaccine or infectious disease research in general and for research assay validation.

Stored samples may also be used later for evaluation of exploratory genetic factors that may influence the immune response to vaccination. For example, this could include RNA transcriptome, immunoglobulin genes, and polymorphisms, in pattern recognition receptors (PRRs), such as Toll-like receptor (TLR) or RIG-like receptor (RLR) genes. In addition, it could include many single-nucleotide polymorphisms (SNPs) in other genes, for example, those coding for cytokines, viral, or vitamin receptors that are experimentally associated with variations in vaccine responses. Other optional analysis, including proteome, lipidome, metabolome, and exosome may be done on collected specimens to evaluate some proteins, lipids, metabolites, and low molecular weight molecules involved in the immune response to vaccination.

No specific results will be provided to participants or their health care providers because we will not be investigating genetic analyses that have known medical diagnoses (e.g. Huntington's disease) or other medically actionable genetic information.

No personal identifiable information will be shared since the results will only be shared with a code.

8.6.2 Storage and Tracking of Blood Samples and Other Specimens

All of the stored study research samples are labeled by a code that only the site can link to the subject. Samples are stored at the VIP, Gaithersburg, MD or VRC laboratories in Building 40, Bethesda, MD, which are both secure facilities with limited access. Data will be kept in password-protected computers. Only investigators or their designees will have access to the samples and data. Samples will be tracked in the Laboratory Information Management System (LIMS) database or using another software designed for this purpose (e.g., Freezerworks).

8.6.3 Disposition of Samples, Specimens and Data at Completion of the Protocol

In the future, other investigators (both at NIH and outside) may wish to study these samples and/or data. IRB approval must be sought prior to any sharing of samples. Any clinical information shared about those samples would similarly require prior IRB approval. The research use of stored, unlinked or unidentified samples may be exempt from the need for prospective IRB review and approval.

At the time of protocol termination, samples will remain in the VIP facility or VRC laboratories or, after IRB approval, transferred to another repository. Regulatory oversight of the stored samples and data may be transferred to a stored samples protocol as part of the IRB-approved termination plan. Data will be archived by the VRC/DMID in compliance with requirements for

retention of research records, or after IRB and study sponsor approval, it may be either destroyed or transferred to another repository.

8.6.4 Loss or Destruction of Samples, Specimens or Data

Any loss or unanticipated destruction of samples (for example, due to freezer malfunction) or data (for example, misplacing a printout of data with identifiers) that compromises the scientific integrity of the study will be reported to the IRB in accordance with institutional policies. The PI will also notify the IRB if the decision is made to destroy the remaining samples.

8.7 Safety Oversight

8.7.1 Protocol Safety Review Team

Close cooperation between the designated members of the Protocol Team will occur to evaluate and respond to individual AEs in a timely manner. The VRC designated Safety Officer conducts a daily safety review of clinical data per VRC Standard Operating Procedures.

The PSRT is comprised of the PI, Associate Investigators, Study Coordinator, Protocol Specialists, and other study clinicians. In addition, PSRT may include IND Sponsor representatives, the DMID Medical Monitor (MM), representative(s) from the product manufacturer, and an independent medical expert as needed.

PSRT will review the summary study safety data reports on a weekly basis through 4 weeks after the last subject receives the final study injection. After this time, the PSRT will monitor the safety data reports on a monthly basis through completion of the last study visit.

8.7.2 Safety Monitoring Committee (SMC)

The SMC is an independent group of at least 3 experts that monitors subject safety and advises DMID. SMC members will be separate and independent of study personnel participating in this trial and should not have scientific, financial, or other conflicts of interest related to this trial. The SMC will consist of members with appropriate expertise to contribute to the interpretation of data from this trial. A quorum will consist of a simple majority.

The SMC will hold an organizational meeting prior to enrollment. At this meeting, the SMC will review the charter, protocol, ICF, and safety report template.

For periodic e-review and evaluation of the accumulated study data for participant safety, study conduct, and progress, the SMC will receive the following:

- Tabulated AEs and study characteristics (demographics, enrollment, etc) based on data through day 15 after each cohort is enrolled;
- All SAEs, AESI and MAAEs.

After the organizational meeting, no routine meetings of the SMC are needed. The SMC will conduct ad hoc meetings for the following:

- As requested by the SMC (based on AE or SAEs);
- When trial-level halting criteria are met;
- At the request of DMID to review a potential safety concern identified by either the PI, DMID Medical Monitor, or Protocol Safety Review Team (PSRT).

Procedures for SMC reviews/meetings are defined in the SMC charter. If ad hoc meetings are needed, the SMC will review applicable data including, but not limited to, enrollment, demographics, dosing, data, laboratory data and safety data at scheduled time points during this trial as defined in the SMC charter. Additional data may be requested by the SMC. As an outcome of each review/meeting, the SMC will make a recommendation as to the advisability of proceeding with study drug administration, and to continue, modify, or terminate this trial.

Once approved by the Chair, the SMC recommendations will be forwarded to the DMID Clinical Project Manager and MM for their review and approval and inclusion of action items for implementation, as applicable. The SMC final signed recommendations will be distributed to the SMC Members, the investigator(s), and the Study Team within three (3) business days.

9. ADMINISTRATIVE AND OPERATIONAL OBLIGATIONS

9.1 Protocol Amendments

Protocol amendments must be made only with prior approval of the IND Sponsor and with agreement from the PI and MO. All study amendments will be submitted to the IRB for approval.

The IND Sponsor, the IRB, OHRP, the PI, Protocol Chairs, and/or the FDA reserve the right to terminate the study. The PI will notify the IRB in writing of the study's completion or early termination.

9.2 Study Documentation and Storage

The PI will delegate the study responsibilities to the study team, and a list of appropriately qualified persons to whom trial duties have been delegated will be maintained.

Source documents are original data, records, and other information associated with and concerning the subject. These include but are not limited to hospital records, clinical and office charts, laboratory and pharmacy records, and correspondence. Long-term storage of source documents may be in the form of electronic files.

The PI and staff are responsible for maintaining a comprehensive and centralized filing system of all study-related (essential) documentation, suitable for inspection at any time by representatives of the IND Sponsor (DMID), the VRC/NIAID/NIH, IRB, NIH, FDA, and/or applicable regulatory authorities. Elements include:

1. Subject files containing completed informed consent forms and supporting copies of source documentation.
2. Study files containing the protocol with all amendments, IBs, copies of all correspondence with the IRB.

In addition, all original source documentation must be maintained and be readily available.

All essential documentation should be retained by the institution for the same period of time required for medical records retention. The FDA requires study records to be retained for up to three years after marketing approval or refusal (21 CFR 312.62). If no marketing application is filed, or if the application is not approved, the records will be retained for two years after the investigation is discontinued and the FDA is notified. The HHS protection of human subjects' regulations require that institutions retain records of IRB/EC activities and documentation of informed consent of subjects for at least 3 years after study completion (45 CFR 46).

No study document should be destroyed without prior written agreement between the VRC, DMID and the investigator. Should the investigator wish to assign the study records to another party or move them to another location, they must notify the VRC in writing of the new responsible person and/or the new location.

9.3 Clinical Monitoring

Clinical site monitoring is conducted to ensure that the rights and well-being of trial participants are protected, that the reported trial data are accurate, complete, and verifiable, and that the conduct of the trial is in compliance with the currently approved protocol/amendment(s), with ICH GCP, and with applicable regulatory requirement(s).

Monitoring for this study will be performed as designated by the IND Sponsor, DMID. DMID may collaborate with the VRC Clinical Monitoring Oversight Group as needed.

9.4 Data Collection and Data Sharing

9.4.1 Data Collection

Clinical research data will be collected in a secure electronic web-based clinical data management system (CDMS) through a CRO, The Emmes Company, LLC (Rockville, MD). Extracted data without patient identifiers will be sent to the Protocol Statistician for statistical analysis.

9.4.2 Source Documents

The site will maintain appropriate medical and research records for this trial, in compliance with ICH E6(R2) GCP, applicable regulations, and institutional requirements for the protection of confidentiality of subjects. Source data are all information, original records of clinical findings, observations, or other activities in a clinical trial necessary for the reconstruction and evaluation of the trial. Examples of these original documents and data records include, but are not limited to, medical records, laboratory reports, pharmacy records and other research records maintained for the clinical trial.

9.4.3 Data Sharing Plan

Data generated in this study will be shared as de-identified data in the government-funded public repository, www.ClinicalTrials.gov. Data may be shared prior to publication at approved public presentations or for collaborative development and will be shared at the time of publication or within 1 year of the primary completion date.

9.5 Quality Assurance and Quality Control

The clinical site will perform internal quality management of study conduct, data and biological specimen collection, documentation and completion. The VEC's Quality Management Plan will be used to perform quality management for this trial.

Quality control (QC) procedures will be implemented beginning with the data entry system and data QC checks that will be run on the database will be generated. Any missing data or data anomalies will be communicated to the site for clarification/resolution.

The monitors will verify that the clinical trial is conducted, and data are generated, and biological specimens are collected, documented (recorded), and reported in compliance with the protocol, ICH GCP, and applicable regulatory requirements.

9.6 Language

All written information and other material to be used by subjects and investigative staff must use vocabulary and language that are clearly understood by the subject.

9.7 Research-Related Injuries

The NIH CC will provide short-term medical care for any injury resulting from participation in this research. In general, the NIH, the NIH CC, or the U.S. Federal Government will provide no long-term medical care or financial compensation for research-related injuries.

10. REFERENCES

1. Marsh, G.A., et al., *Cedar virus: a novel Henipavirus isolated from Australian bats.* PLoS Pathog, 2012. **8**(8): p. e1002836.
2. Harcourt, B.H., et al., *Genetic characterization of Nipah virus, Bangladesh, 2004.* Emerg Infect Dis, 2005. **11**(10): p. 1594-7.
3. Goh, K.J., et al., *Clinical features of Nipah virus encephalitis among pig farmers in Malaysia.* N Engl J Med, 2000. **342**(17): p. 1229-35.
4. Chadha, M.S., et al., *Nipah virus-associated encephalitis outbreak, Siliguri, India.* Emerg Infect Dis, 2006. **12**(2): p. 235-40.
5. Arankalle, V.A., et al., *Genomic characterization of Nipah virus, West Bengal, India.* Emerg Infect Dis, 2011. **17**(5): p. 907-9.
6. Arunkumar, G., et al., *Outbreak Investigation of Nipah Virus Disease in Kerala, India, 2018.* J Infect Dis, 2019. **219**(12): p. 1867-1878.
7. Gurley, E.S., et al., *Person-to-person transmission of Nipah virus in a Bangladeshi community.* Emerg Infect Dis, 2007. **13**(7): p. 1031-7.
8. Luby, S.P., et al., *Recurrent zoonotic transmission of Nipah virus into humans, Bangladesh, 2001-2007.* Emerg Infect Dis, 2009. **15**(8): p. 1229-35.
9. Sweileh, W.M., *Global research trends of World Health Organization's top eight emerging pathogens.* Global Health, 2017. **13**(1): p. 9.
10. Clayton, B.A., et al., *The Nature of Exposure Drives Transmission of Nipah Viruses from Malaysia and Bangladesh in Ferrets.* PLoS Negl Trop Dis, 2016. **10**(6): p. e0004775.
11. Mire, C.E., et al., *Pathogenic Differences between Nipah Virus Bangladesh and Malaysia Strains in Primates: Implications for Antibody Therapy.* Sci Rep, 2016. **6**: p. 30916.
12. Clayton, B.A., et al., *Transmission routes for nipah virus from Malaysia and Bangladesh.* Emerg Infect Dis, 2012. **18**(12): p. 1983-93.
13. Sazzad, H.M., et al., *Nipah virus infection outbreak with nosocomial and corpse-to-human transmission, Bangladesh.* Emerg Infect Dis, 2013. **19**(2): p. 210-7.
14. Chua, K.B., et al., *Fatal encephalitis due to Nipah virus among pig-farmers in Malaysia.* Lancet, 1999. **354**(9186): p. 1257-9.
15. Hsu, V.P., et al., *Nipah virus encephalitis reemergence, Bangladesh.* Emerg Infect Dis, 2004. **10**(12): p. 2082-7.
16. Abdullah, S. and C.T. Tan, *Henipavirus encephalitis.* Handb Clin Neurol, 2014. **123**: p. 663-70.
17. Lo, M.K. and P.A. Rota, *The emergence of Nipah virus, a highly pathogenic paramyxovirus.* J Clin Virol, 2008. **43**(4): p. 396-400.
18. Prescott, J., et al., *The immune response to Nipah virus infection.* Arch Virol, 2012. **157**(9): p. 1635-41.
19. Wong, K.T. and C.T. Tan, *Clinical and pathological manifestations of human henipavirus infection.* Curr Top Microbiol Immunol, 2012. **359**: p. 95-104.
20. Ching, P.K., et al., *Outbreak of henipavirus infection, Philippines, 2014.* Emerg Infect Dis, 2015. **21**(2): p. 328-31.
21. AbuBakar, S., et al., *Isolation and molecular identification of Nipah virus from pigs.* Emerg Infect Dis, 2004. **10**(12): p. 2228-30.

22. Ang, B.S.P., T.C.C. Lim, and L. Wang, *Nipah Virus Infection.* J Clin Microbiol, 2018. **56**(6).
23. Chua, K.B., *Risk factors, prevention and communication strategy during Nipah virus outbreak in Malaysia.* Malays J Pathol, 2010. **32**(2): p. 75-80.
24. Field, H., et al., *The natural history of Hendra and Nipah viruses.* Microbes Infect, 2001. **3**(4): p. 307-14.
25. Geisbert, T.W., H. Feldmann, and C.C. Broder, *Animal challenge models of henipavirus infection and pathogenesis.* Curr Top Microbiol Immunol, 2012. **359**: p. 153-77.
26. Glennon, E.E., et al., *Domesticated animals as hosts of henipaviruses and filoviruses: A systematic review.* Vet J, 2018. **233**: p. 25-34.
27. Yob, J.M., et al., *Nipah virus infection in bats (order Chiroptera) in peninsular Malaysia.* Emerg Infect Dis, 2001. **7**(3): p. 439-41.
28. Donaldson, H. and D. Lucey, *Enhancing preparation for large Nipah outbreaks beyond Bangladesh: Preventing a tragedy like Ebola in West Africa.* Int J Infect Dis, 2018. **72**: p. 69-72.
29. Halpin, K., et al., *Pteropid bats are confirmed as the reservoir hosts of henipaviruses: a comprehensive experimental study of virus transmission.* Am J Trop Med Hyg, 2011. **85**(5): p. 946-51.
30. Rahman, S.A., et al., *Characterization of Nipah virus from naturally infected Pteropus vampyrus bats, Malaysia.* Emerg Infect Dis, 2010. **16**(12): p. 1990-3.
31. Yadav, P.D., et al., *Detection of Nipah virus RNA in fruit bat (Pteropus giganteus) from India.* Am J Trop Med Hyg, 2012. **87**(3): p. 576-8.
32. Bloom, D.E. and D. Cadarette, *Infectious Disease Threats in the Twenty-First Century: Strengthening the Global Response.* Front Immunol, 2019. **10**: p. 549.
33. Loomis, R.J., et al., *Structure-Based Design of Nipah Virus Vaccines: A Generalizable Approach to Paramyxovirus Immunogen Development.* Front Immunol, 2020. **11**: p. 842.
34. Loomis, R.J., et al., *Chimeric Fusion (F) and Attachment (G) Glycoprotein Antigen Delivery by mRNA as a Candidate Nipah Vaccine.* Front Immunol, 2021. **12**: p. 772864.
35. Baden, L.R., et al., *Efficacy and Safety of the mRNA-1273 SARS-CoV-2 Vaccine.* N Engl J Med, 2021. **384**(5): p. 403-416.
36. Polack, F.P., et al., *Safety and Efficacy of the BNT162b2 mRNA Covid-19 Vaccine.* N Engl J Med, 2020. **383**(27): p. 2603-2615.
37. Anderson, E.J., et al., *Safety and Immunogenicity of SARS-CoV-2 mRNA-1273 Vaccine in Older Adults.* N Engl J Med, 2020. **383**(25): p. 2427-2438.
38. Jackson, L.A., et al., *An mRNA Vaccine against SARS-CoV-2 - Preliminary Report.* N Engl J Med, 2020. **383**(20): p. 1920-1931.
39. Corbett, K.S., et al., *SARS-CoV-2 mRNA vaccine design enabled by prototype pathogen preparedness.* Nature, 2020. **586**(7830): p. 567-571.
40. Corbett, K.S., et al., *Evaluation of the mRNA-1273 Vaccine against SARS-CoV-2 in Nonhuman Primates.* N Engl J Med, 2020. **383**(16): p. 1544-1555.
41. Corbett, K.S., et al., *Immune Correlates of Protection by mRNA-1273 Immunization against SARS-CoV-2 Infection in Nonhuman Primates.* bioRxiv, 2021.
42. Vogel, A.B., et al., *BNT162b vaccines protect rhesus macaques from SARS-CoV-2.* Nature, 2021. **592**(7853): p. 283-289.

43. Laczko, D., et al., *A Single Immunization with Nucleoside-Modified mRNA Vaccines Elicits Strong Cellular and Humoral Immune Responses against SARS-CoV-2 in Mice.* Immunity, 2020. **53**(4): p. 724-732 e7.
44. Lederer, K., et al., *SARS-CoV-2 mRNA Vaccines Foster Potent Antigen-Specific Germinal Center Responses Associated with Neutralizing Antibody Generation.* Immunity, 2020. **53**(6): p. 1281-1295 e5.
45. Wrapp, D., et al., *Cryo-EM structure of the 2019-nCoV spike in the prefusion conformation.* Science, 2020. **367**(6483): p. 1260-1263.
46. Ruggeberg, J.U., et al., *Anaphylaxis: case definition and guidelines for data collection, analysis, and presentation of immunization safety data.* Vaccine, 2007. **25**(31): p. 5675-84.
47. Aretz, H.T., et al., *Myocarditis. A histopathologic definition and classification.* Am J Cardiovasc Pathol, 1987. **1**(1): p. 3-14.
48. Gargano, J.W., et al., *Use of mRNA COVID-19 Vaccine After Reports of Myocarditis Among Vaccine Recipients: Update from the Advisory Committee on Immunization Practices - United States, June 2021.* MMWR Morb Mortal Wkly Rep, 2021. **70**(27): p. 977-982.
49. Ferreira, V.M., et al., *Cardiovascular Magnetic Resonance in Nonischemic Myocardial Inflammation: Expert Recommendations.* Journal of the American College of Cardiology, 2018. **72**(24): p. 3158-3176.
50. Adler, Y., et al., *2015 ESC Guidelines for the diagnosis and management of pericardial diseases: The Task Force for the Diagnosis and Management of Pericardial Diseases of the European Society of Cardiology (ESC)Endorsed by: The European Association for Cardio-Thoracic Surgery (EACTS).* European Heart Journal, 2015. **36**(42): p. 2921-2964.

APPENDIX I: SCHEDULE OF EVALUATIONS

		VRC 500	VRC 322/DMID 20-0016: Group 1, Group 2, Group 3 and Group 4 (25, 50, 100 and 10 mcg of mRNA-1215 IM, respectively)														
**Visit Number**		*01	02	02A	03	04	05	05A	06	07	08	09	12	14	16	18	20
**Week of Study**		-8 to 0	W0	W1	W1	W2	W4	W4	W5	W6	W8	W12	W16	W24	W32	W44	W56
**Day of Study**		-56 to 0	[1]D0	D1	D7	D14	D28	D29	D35	D42	D56	D84	D112	D168	D224	D308	D392
**Clinical**	Tube^																
*VRC 500 Screening Consent		X															
VRC 322/DMID 21-0016 AoU; Consent			X														
[2]Physical exam		X	X		X	X	X		X	X	X	X	X	X	X	X	X
[3]Medical history		X	X		X	X	X		X	X	X	X	X	X	X	X	X
[4]Study Product Administration			**X**				**X**										
Phone evaluation (clinic visit as needed)				X				X									
Begin diary card			X				X										
[5]Pregnancy test: urine or serum		X	X				X			[6][X]						X	
[5]Pregnancy Prevention Counseling/Reproductive Form		X	X				X			[6][X]						X	
CBC with differential	EDTA	3	3		3		3		3		3					3	
Total bilirubin, AST, ALT, ALP	GLT	4	4		4		4		4		4					4	
Creatinine		X	X		X		X		X		X					X	
HIV (other tests, if needed)	EDTA	3															
**Research Samples**																	
Serum	SST	16	16	-	8	8	16	-	8	16	8	8	8	8	8	8	8
PBMC and plasma	EDTA	20	40	-	20	20	40	-	80	[7]120 or apheresis	20	40	40	40	40	40	40
**Daily Volume (mL)**		46	63	0	35	28	63	0	95	136	35	48	48	48	48	55	48
**Max. Cumulative Volume (mL)**		46	109	109	144	172	235	235	330	466	501	549	597	645	693	748	796

Visit windows: Schedule Visits 02A – 5 with respect to Visit 02 and Visits 05A – 20 with respect to Visit 05 per the following visit windows:

Visits 02A, 05A (+1 day). Visit 03 and 06 (±1 day). Visits 04, 05, 07 (± 2 days). Visits 08, 09, 12, 14, 16, 18, 20 (±7 days).

Footnotes continue to next page

* VRC 500: Screening evaluations must be no more than 56 days prior to Day 0 to be used for eligibility (negative pregnancy test from Day 0 must be used for eligibility). If clinical assessment on Day 0 suggests significant changes may have occurred since screening, then physical examination & clinical laboratory studies done on Day 0 are used for eligibility. Research blood samples may be collected at any time during screening through enrollment and are not to be restricted to the 56-day prior to enrollment.

^ Tube: Tube types and volumes are shown to meet current institutional requirements and projected blood volumes. Different tubes for clinical evaluations may be used to meet site requirements. Slightly different volumes of tubes may be used for research blood samples when tube sizes as shown are not available, or as otherwise instructed by the IND Sponsor. Samples for safety and primary immunogenicity evaluations should be collected first, and other research samples as permitted with adequate supplies. Collected blood volumes will stay within the NIH CC blood draw limits for each subject.

[1] Day 0=day of enrollment and vaccine injection. Day 0 evaluations prior to injection are the baseline for assessing adverse events subsequently.

[2] Screening visit includes physical exam with vital signs. Weight should be measured at screening and on Visit 02. At other visits, physical exam is done if indicated. Otherwise, only blood pressure (BP), pulse, temperature, and respiration are required.

[3] Perform medical history targeted to eligibility at screening, then interim medical history

[4] Study Product Administration: Complete post vaccination evaluations (BP, pulse, temperature, respiration and injection site assessment) at 30 minutes or longer after injection.

[5] Negative pregnancy test results must be confirmed for women of reproductive potential prior to study injection.

[6] For women of reproductive potential, pregnancy test must be negative within 72 hours prior to apheresis procedure. Brackets [ ] indicate optional as needed for those who complete apheresis.

[7] If optional apheresis occurs, ONLY draw 16 mL in SST (DO NOT draw 120 ml in EDTA tubes)

		VRC 500	VRC 322/DMID 20-0016: Subjects with discontinued vaccination schedule**										
**Visit Number**		*01	02	02A	03	04	05	08	09	13	15	17	19
**Week of Study**		-8 to 0	W0	W1	W1	W2	W4	W8	W12	W20	W28	W40	W52
**Day of Study**		-56 to 0	[1]D0	D1	D7	D14	D28	D56	D84	D140	D196	D280	D364
**Clinical**	Tube												
*VRC 500 Screening Consent		X											
VRC 322/DMID 21-0016 AoU; Consent			X										
[2]Physical exam		X	X		X	X	X	X	X	X	X	X	X
[3]Medical history		X	X		X	X	X	X	X	X	X	X	X
[4]Study Product Administration			**X**										
Phone evaluation (clinic visit as needed)				X									
Begin diary card			X										
[5]Pregnancy test: urine or serum		X	X			[6][X]						X	
[5]Pregnancy Prevention Counseling/Reproductive Form		X	X			[6][X]						X	
CBC with differential	EDTA	3	3		3		3					3	
Total bilirubin, AST, ALT, ALP	GLT	4	4		4		4					4	
Creatinine		X	X		X		X					X	
HIV (other tests, if needed)	EDTA	3											
**Research Samples**													
Serum	SST	16	16	-	16	16	8	8	8	8	8	8	8
PBMC and plasma	EDTA	20	40	-	80	[7]120 or apheresis	40	40	40	40	40	40	40
**Daily Volume (mL)**		46	63	0	103	136	55	48	48	48	48	55	48
**Max. Cumulative Volume (mL)**		46	109	109	212	348	403	451	499	547	595	650	698

**This is a suggested schedule for subjects with discontinued product administration that can be followed by PI or designee discretion

Footnotes continue to next page

Visit windows: Schedule Visits 02A – 19 with respect to Visit 02 per the following visit windows:
Visits 02A (+1 day). Visit 03 (±1 day). Visits 04, 05 (±2 days). Visits 08, 09, 13, 15, 17, 19 (±7 days).

* VRC 500: Screening evaluations must be no more than 56 days prior to Day 0 to be used for eligibility (negative pregnancy test from Day 0 must be used for eligibility). If clinical assessment on Day 0 suggests significant changes may have occurred since screening, then physical examination & clinical laboratory studies done on Day 0 are used for eligibility. Research blood samples may be collected at any time during screening through enrollment and are not to be restricted to the 56-day prior to enrollment.

^ Tube: Tube types and volumes are shown to meet current institutional requirements and projected blood volumes. Different tubes for clinical evaluations may be used to meet site requirements. Slightly different volumes of tubes may be used for research blood samples when tube sizes as shown are not available, or as otherwise instructed by the IND Sponsor. Samples for safety and primary immunogenicity evaluations should be collected first, and other research samples as permitted with adequate supplies. Collected blood volumes will stay within the NIH CC blood draw limits for each subject.

[1] Day 0=day of enrollment and vaccine injection. Day 0 evaluations prior to injection are the baseline for assessing adverse events subsequently.

[2] Screening visit includes physical exam with vital signs. Weight should be measured at screening and on Visit 02. At other visits, physical exam is done if indicated. Otherwise, only blood pressure (BP), pulse, temperature, and respiration are required.

[3] Perform medical history targeted to eligibility at screening, then interim medical history

[4] Study Product Administration: Complete post vaccination evaluations (BP, pulse, temperature, respiration and injection site assessment) at 30 minutes or longer after injection.

[5] Negative pregnancy test results must be confirmed for women of reproductive potential prior to study injection.

[6] For women of reproductive potential, pregnancy test must be negative within 72 hours prior to apheresis procedure. Brackets [ ] indicate optional as needed for those who complete apheresis.

[7] If optional apheresis occurs, ONLY draw 16 mL in SST (DO NOT draw 120 ml in EDTA tubes)

APPENDIX II: ASSESSMENT OF RELATIONSHIP TO VACCINE AND GRADING SEVERITY OF ADVERSE EVENTS

Assessment of Relationship of an Adverse Event to Study Vaccine:

The relationship between an AE and the vaccine will be assessed by the investigator on the basis of his or her clinical judgment and the definitions below.

- **Definitely Related.** The AE and administration of study agent are related in time, and a direct association can be demonstrated.
- **Probably Related**. The AE and administration of study agent are reasonably related in time, and the AE is more likely explained by study agent than other causes.
- **Possibly Related.** The AE and administration of study agent are reasonably related in time, but the AE can be explained equally well by causes other than study agent.
- **Not Related.** The AE is clearly explained by another cause not related to the study product.

For purposes of preparing summary data reports in which AE attributions are simplified to "Related" or "Not Related", in this protocol, the "Definitely, Probably and Possibly" attributions above will be mapped to the "Related" category while the "Unlikely/Probably Not Related" and "Not Related" attributions above will be mapped to the "Not Related" category. The definitions that apply when these two attribution categories alone are used are as follows:

- **Related** – There is a reasonable possibility that the AE may be related to the study product(s).
- **Not Related** – There is not a reasonable possibility that the AE is related to the study product(s).

Grading the Severity of Adverse Events:

The FDA Guidance for Industry (September 2007): "Toxicity Grading Scale for Healthy Adult and Adolescent Volunteers Enrolled in Preventive Vaccine Clinical Trials" is the basis for the severity grading of AEs in this protocol. Several modifications were made to the table as follows:

- "Emergency room visit" is not automatically considered a life-threatening event; these words have been removed from any "Grade 4" definition where they appear in the table copied from the guidance document.
- Laboratory value shown as a "graded" value in the table that is within the institutional normal range will not be severity graded or recorded as an AE.
- Severity grading for hemoglobin decrease on the basis of the magnitude of decrease from baseline is not applicable at the Grade 1 level; only absolute hemoglobin will be used to define Grade 1.
- Severity grading for Grade 4 local reaction to injectable product (Erythema/Redness and Induration/Swelling) refer to necrosis or exfoliative dermatitis "requiring medical attention."
- Bruising or skin lesion associated with study injection will be assessed using the same severity grading as for erythema/redness.

When not otherwise specified, the following guidance will be used to assign a severity grade:

- **Grade 1 (Mild)**: No effect on activities of daily living
- **Grade 2 (Moderate)**: Some interference with activity not requiring medical intervention
- **Grade 3 (Severe)**: Prevents daily activity and requires medical intervention
- **Grade 4 (Potentially Life-threatening)**: Hospitalization; immediate medical intervention or therapy required to prevent death.
- **Grade 5 (Death)**: Death is assigned a Grade 5 severity. Only the single AE that is assessed as the primary cause of death should be assigned "Grade 5" severity.

Toxicity Grading Scale for Healthy Adult and Adolescent Volunteers Enrolled in Preventive Vaccine Clinical Trials

Modified from FDA Guidance - September 2007

A. Tables for Clinical Abnormalities

**Local Reaction to Injectable Product**	**Mild (Grade 1)**	**Moderate (Grade 2)**	**Severe (Grade 3)**	**Potentially Life Threatening (Grade 4)**
Pain	Does not interfere with activity	Repeated use of non-narcotic pain reliever > 24 hours or interferes with activity	Any use of narcotic pain reliever or prevents daily activity	Hospitalization
Tenderness	Mild discomfort to touch	Discomfort with movement	Significant discomfort at rest	Hospitalization
[1,2]Erythema/Redness	2.5 – 5 cm	5.1 – 10 cm	> 10 cm	Necrosis or exfoliative dermatitis requiring medical attention
[3]Induration/Swelling	2.5 – 5 cm and does not interfere with activity	5.1 – 10 cm or interferes with activity	> 10 cm or prevents daily activity	Necrosis requiring medical attention
*Axillary (underarm) swelling or tenderness ipsilateral to the site of injection	Does not interfere with activity	Repeated use of non-narcotic pain reliever > 24 hours or interferes with activity	Any use of narcotic pain reliever or prevents daily activity	Hospitalization
* Added to the FDA guidance to collect lymphadenopathy symptoms				

[4] Vital Signs	Mild (Grade 1)	Moderate (Grade 2)	Severe (Grade 3)	Potentially Life Threatening (Grade 4)
[5]Fever (°C)<br>(°F)	38.0 – 38.4<br>100.4 – 101.1	38.5 – 38.9<br>101.2 – 102.0	39.0 – 40<br>102.1 – 104	> 40<br>> 104
Tachycardia - beats per minute	101 – 115	116 – 130	> 130	Hospitalization for arrhythmia
[6]Bradycardia - beats per Minute	50 – 54	45 – 49	< 45	Hospitalization for arrhythmia
Hypertension (systolic) - mm Hg	141 – 150	151 – 155	> 155	Hospitalization for malignant hypertension
Hypertension (diastolic) - mm Hg	91 – 95	96 – 100	> 100	Hospitalization for malignant hypertension
Hypotension (systolic) – mm Hg	85 – 89	80 – 84	< 80	Hospitalization for hypotensive shock
Respiratory Rate – breaths per minute	17 – 20	21 – 25	> 25	Intubation

[1]In addition to grading the measured local reaction at the greatest single diameter, the measurement should be recorded as a continuous variable.
[2]Bruising or skin lesion associated with study injection will be assessed using the same severity grading as for erythema/redness.
[3]Induration/Swelling should be evaluated and graded using the functional scale as well as the actual measurement.
[4]Subject should be at rest for all vital sign measurements.
[5]Oral temperature; no recent hot or cold beverages or smoking.
[6]When resting heart rate is between 60 – 100 beats per minute. Use clinical judgment when characterizing Bradycardia among some healthy subject populations, for example, conditioned athletes.

**Systemic (General)**	**Mild (Grade 1)**	**Moderate (Grade 2)**	**Severe (Grade 3)**	**Potentially Life Threatening (Grade 4)**
Nausea/vomiting	No interference with activity or 1 – 2 episodes/24 hours	Some interference with activity or > 2 episodes/24 hours	Prevents daily activity, requires outpatient IV hydration	Hospitalization for hypotensive shock
Diarrhea	2 – 3 loose stools or < 400 gms/24 hours	4 – 5 stools or 400 – 800 gms/24 hours	6 or more watery stools or > 800gms/24 hours or requires outpatient IV hydration	Hospitalization
Headache	No interference with activity	Repeated use of non-narcotic pain reliever > 24 hours or some interference with activity	Significant; any use of narcotic pain reliever or prevents daily activity	Hospitalization
Fatigue	No interference with activity	Some interference with activity	Significant; prevents daily activity	Hospitalization
Myalgia	No interference with activity	Some interference with activity	Significant; prevents daily activity	Hospitalization
Illness or clinical adverse event (as defined according to applicable regulations)	No interference with activity	Some interference with activity not requiring medical intervention	Prevents daily activity and requires medical intervention	Hospitalization

B. Tables for Laboratory Abnormalities

The laboratory values provided in the tables below serve as guidelines and are dependent upon the institutional normal parameters. Institutional normal reference ranges should be provided to demonstrate that they are appropriate.

Serum*	Mild (Grade 1)	Moderate (Grade 2)	Severe (Grade 3)	Potentially Life Threatening (Grade 4) **
Sodium – Hyponatremia mEq/L	132 – 134	130 – 131	125 – 129	< 125
Sodium – Hypernatremia mEq/L	144 – 145	146 – 147	148 – 150	> 150
Potassium – Hyperkalemia mEq/L	5.1 – 5.2	5.3 – 5.4	5.5 – 5.6	> 5.6
Potassium – Hypokalemia mEq/L	3.5 – 3.6	3.3 – 3.4	3.1 – 3.2	< 3.1
Glucose – Hypoglycemia mg/dL	65 – 69	55 – 64	45 – 54	< 45
Glucose – Hyperglycemia<br>Fasting – mg/dL<br>Random – mg/dL	 100 – 110<br>110 – 125	 111 – 125<br>126 – 200	 >125<br>>200	Insulin requirements or hyperosmolar coma
Blood Urea Nitrogen BUN mg/dL	23 – 26	27 – 31	> 31	Requires dialysis
Creatinine – mg/dL	1.5 – 1.7	1.8 – 2.0	2.1 – 2.5	> 2.5 or requires dialysis
Calcium – hypocalcemia mg/dL	8.0 – 8.4	7.5 – 7.9	7.0 – 7.4	< 7.0
Calcium – hypercalcemia mg/dL	10.5 – 11.0	11.1 – 11.5	11.6 – 12.0	> 12.0
Magnesium – hypomagnesemia mg/dL	1.3 – 1.5	1.1 – 1.2	0.9 – 1.0	< 0.9
Phosphorous – hypophosphatemia mg/dL	2.3 – 2.5	2.0 – 2.2	1.6 – 1.9	< 1.6
CPK – mg/dL	1.25 – 1.5 x ULN***	>1.5 – 3.0 x ULN	>3.0 –10 x ULN	> 10 x ULN
Albumin – Hypoalbuminemia g/dL	2.8 – 3.1	2.5 – 2.7	< 2.5	--
Total Protein – Hypoproteinemia g/dL	5.5 – 6.0	5.0 – 5.4	< 5.0	--
Alkaline phosphate – increase by factor	1.1 – 2.0 x ULN	2.1 – 3.0 x ULN	> 3.0 – 10 x ULN	> 10 x ULN

Serum*	Mild (Grade 1)	Moderate (Grade 2)	Severe (Grade 3)	Potentially Life Threatening (Grade 4) **
Liver Function Tests – ALT, AST increase by factor	1.1 – 2.5 x ULN	> 2.6 – 5.0 x ULN	> 5.1 – 10 x ULN	> 10 x ULN
Bilirubin – when accompanied by any increase in Liver Function Test increase by factor	1.1 – 1.25 x ULN	> 1.26 – 1.5 x ULN	> 1.51 – 1.75 x ULN	> 1.75 x ULN
Bilirubin – when Liver Function Test is normal; increase by factor	1.1 – 1.5 x ULN	1.6 – 2.0 x ULN	2.0 – 3.0 x ULN	> 3.0 x ULN
Cholesterol	201 – 210	211 – 225	> 226	---
Pancreatic enzymes – amylase, lipase	1.1 – 1.5 x ULN	1.6 – 2.0 x ULN	2.1 – 5.0 x ULN	> 5.0 x ULN

* The laboratory values provided in the tables serve as guidelines and are dependent upon institutional normal parameters. Institutional normal reference ranges should be provided to demonstrate that they are appropriate.
** The clinical signs or symptoms associated with laboratory abnormalities might result in characterization of the laboratory abnormalities as Potentially Life Threatening (Grade 4). For example, a low sodium value that falls within a Grade 3 parameter (125-129 mE/L) should be recorded as a Grade 4 hyponatremia event if the subject had a new seizure associated with the low sodium value.
***ULN is the upper limit of the normal range.

Hematology *	Mild (Grade 1)	Moderate (Grade 2)	Severe (Grade 3)	Potentially Life Threatening (Grade 4)
Hemoglobin (Female) - gm/dL	11.0 – 12.0	9.5 – 10.9	8.0 – 9.4	< 8.0
Hemoglobin (Female) decrease from baseline value - gm/dL	not applicable	1.6 – 2.0	2.1 – 5.0	> 5.0
Hemoglobin (Male) - gm/dL	12.5 – 13.5	10.5 – 12.4	8.5 – 10.4	< 8.5
Hemoglobin (Male) decrease from baseline value – gm/dL	not applicable	1.6 – 2.0	2.1 – 5.0	> 5.0
WBC Increase - cell/mm^3	10,800 – 15,000	15,001 – 20,000	20,001 – 25, 000	> 25,000
WBC Decrease - cell/mm^3	2,500 – 3,500	1,500 – 2,499	1,000 – 1,499	< 1,000
Lymphocytes Decrease - cell/mm^3	750 – 1,000	500 – 749	250 – 499	< 250
Neutrophils Decrease - cell/mm^3	1,500 – 2,000	1,000 – 1,499	500 – 999	< 500
Eosinophils - cell/mm^3	650 – 1500	1501 - 5000	> 5000	Hypereosinophilic
Platelets Decreased - cell/mm^3	125,000 – 140,000	100,000 – 124,000	25,000 – 99,000	< 25,000
PT – increase by factor (prothrombin time)	1.10 x ULN**	> 1.11 – 1.20 x ULN	1.21 – 1.25 x ULN	> 1.25 ULN
PTT – increase by factor (partial thromboplastin time)	1.10 – 1.20 x ULN	1.21 – 1.4 x ULN	1.4 – 1.5 x ULN	> 1.5 x ULN
Fibrinogen increase - mg/dL	400 – 500	501 – 600	> 600	--
Fibrinogen decrease - mg/dL	150 – 200	125 – 149	100 – 124	< 100 or associated with gross bleeding or disseminated intravascular coagulation (DIC)

* The laboratory values provided in the tables serve as guidelines and are dependent upon institutional normal parameters. Institutional normal reference ranges should be provided to demonstrate that they are appropriate.
**ULN is the upper limit of the normal range.

APPENDIX III: ADVERSE EVENTS OF SPECIAL INTEREST TERMS

Investigators should report all events that fall into the categories presented in Table 6 as an AESI per the reporting processes in Section 5.4. These AESIs are medical concepts that are generally of interest in vaccine safety surveillance as per the Brighton Collaboration and Safety Platform for Emergency Vaccines Adverse Events of Special Interest.

Table 6: AESI Categories

Medical Concept	Additional Notes
Thrombocytopenia	• Decreased platelet count that is clinically significant as determined by the PI or designee<br>Including but not limited to immune thrombocytopenia, platelet production decreased, thrombocytopenic purpura, thrombotic thrombocytopenic purpura, or hemolysis, elevated liver enzymes, and low platelet count (HELLP) syndrome
New onset of or worsening of the following neurologic diseases:	• Guillain Barre Syndrome<br>• Acute disseminated encephalomyelitis (ADEM)<br>• Idiopathic peripheral facial nerve palsy (Bell's palsy)<br>• Seizures including but not limited to febrile seizures and/or generalized seizures/convulsions
Anaphylaxis	• Anaphylaxis as defined per protocol<br>• Follow reporting procedures in Section 5.4
Myocarditis/Pericarditis	• Myocarditis<br>• Pericarditis<br>• Myopericarditis

Table 7: Case Definitions of Probable and Confirmed Myocarditis, Pericarditis, and Myopericarditis

<table>
<tr><th>Condition</th><th colspan="2">Definition</th></tr>
<tr><td rowspan="2">Acute myocarditis</td><td>Probable case</td><td>Confirmed case</td></tr>
<tr><td>Presence of ≥ 1 new or worsening of the following clinical symptoms:*
• chest pain, pressure, or discomfort
• dyspnea, shortness of breath, or pain with breathing
• palpitations
• syncope
AND
≥ 1 new finding of
• troponin level above upper limit of normal (any type of troponin)
• abnormal electrocardiogram (ECG or EKG) or rhythm monitoring findings consistent with myocarditis[§]
• abnormal cardiac function or wall motion abnormalities on echocardiogram
• cMRI findings consistent with myocarditis[¶]
AND
• No other identifiable cause of the symptoms and findings</td><td>Presence of ≥ 1 new or worsening of the following clinical symptoms:*
• chest pain, pressure, or discomfort
• dyspnea, shortness of breath, or pain with breathing
• palpitations
• syncope
AND
≥ 1 new finding of
• histopathologic confirmation of myocarditis[†]
• cMRI findings consistent with myocarditis[¶] in the presence of troponin level above upper limit of normal (any type of troponin)
AND
• No other identifiable cause of the symptoms and findings</td></tr>
<tr><td>Acute pericarditis**</td><td colspan="2">Presence of ≥ 2 new or worsening of the following clinical features:
• acute chest pain[††]
• pericardial rub on exam
• new ST-elevation or PR-depression on EKG
• new or worsening pericardial effusion on echocardiogram or MRI</td></tr>
<tr><td>Myopericarditis</td><td colspan="2">This term may be used for patients who meet criteria for both myocarditis and pericarditis.</td></tr>
</table>

Abbreviations: AV = atrioventricular; cMRI = cardiac magnetic resonance imaging; ECG or EKG = electrocardiogram.

* Persons who lack the listed symptoms but who meet other criteria may be classified as subclinical myocarditis (probable or confirmed).
† Using the Dallas criteria [47]. Autopsy cases may be classified as confirmed clinical myocarditis on the basis of meeting histopathologic criteria if no other identifiable cause.
§ To meet the ECG or rhythm monitoring criterion, a probable case must include at least one of 1) ST-segment or T-wave abnormalities; 2) Paroxysmal or sustained atrial, supraventricular, or ventricular arrhythmias; or 3) AV nodal conduction delays or intraventricular conduction defects.
¶ Using either the original or the revised Lake Louise criteria [49].
**[50]
†† Typically described as pain made worse by lying down, deep inspiration, or cough, and relieved by sitting up or leaning forward, although other types of chest pain might occur [48].

PRINCIPAL INVESTIGATOR: Lesia Dropulic, MD

STUDY TITLE: VRC 322/DMID 21-0016: A Phase I, Dose Escalation, Open-Label Clinical Trial to Evaluate Safety, Tolerability and Immunogenicity of a Nipah Virus (NiV) mRNA Vaccine, mRNA-1215, in Healthy Adults

STUDY SITE: NIH/NIAID/VRC/Vaccine Evaluation Clinic (VEC)

Cohort: *Healthy volunteer*

Consent Version: *Version 4.0, December 20, 2023*

WHO DO YOU CONTACT ABOUT THIS STUDY?

Principal Investigator: Lesia Dropulic, MD; [redacted]

Study Coordinator: Laura Novik, RN, MA, CCRC; [redacted]

KEY INFORMATION ABOUT THIS RESEARCH

This consent form describes a research study and is designed to help you decide if you would like to be a part of the research study.

You are being asked to take part in a research study at the National Institutes of Health (NIH). This study is being conducted by the Vaccine Research Center (VRC) and is sponsored by the Division of Microbiology and Infectious Diseases (DMID). This section provides the information we believe is most helpful and important to you in making your decision about taking part in this study. Additional information that may help you decide can be found in other sections of the document. Taking part in research at the NIH is your choice.

This is a study of an experimental vaccine called mRNA-1215 for prevention of the Nipah virus (NiV) infection. The experimental vaccine is a messenger ribonucleic acid (mRNA) vaccine, made of the genetic code of the virus. This genetic code will make a protein from the virus and cause your body to think you have been infected with the virus. It is not made from the Nipah virus and cannot cause Nipah virus infection. This study vaccine has not been given to humans before this study.

The main purpose of this study is to see if the experimental vaccine is safe and how your body responds to it. Since this is the first time we are giving mRNA-1215 to people, we do not know how your body will respond. This study is not designed to protect you from Nipah virus infection.

Nipah virus is transmitted from animals to humans, from human to human and through contaminated food. In infected people, the virus causes mild to severe illness. Some people have no symptoms, some may have respiratory illness, such as cough and difficulty breathing. People with severe symptoms may have brain swelling which can lead to death. Although Nipah virus has caused only a few known outbreaks in Asia, it infects a wide range of animals and can cause

severe disease and death in people, making it a public health concern. There are currently no drugs or vaccines to treat or prevent Nipah virus infection.

About 40 people will take part in this study at the Vaccine Evaluation Clinic (VEC) in the NIH Clinical Center in Bethesda, MD. You will be in the study for about 56 weeks (14 months). You will have about 13 clinic visits during the study. At each clinic visit, you will have about 3 to 14 tubes of blood drawn. Some of your blood will be stored for future research. You will be compensated for your time and efforts for taking part in this study.

You will get 2 doses of the experimental mRNA-1215 vaccine during this study, 1 month apart. The injection (shot) will be given in the upper arm muscle. This is called an intramuscular "IM" injection. We will use a needle and syringe to give you the injection.

You may have side effects from the mRNA-1215 vaccine, such as feeling tired/unwell, fever, muscle aches, headache, chills, nausea, joint pain or injection site reactions including pain/tenderness, redness, swelling, itchiness at the injection site, and lymph node swelling on the same side as the injection. These side effects also commonly occur with FDA approved vaccines. The side effects usually occur within the first 24 hours after the vaccine is given. Rarely, a serious allergic reaction with symptoms like hives, rash, sweating, swelling around the mouth, throat or eyes, wheezing, trouble breathing, increased pulse or sudden weakness may occur shortly after any vaccination. This is called "anaphylaxis" and may be life threatening. Rare cases of myocarditis (inflammation of the heart muscle) and pericarditis (inflammation of the lining around the heart) have been reported in individuals after they received a similar mRNA-based vaccine against COVID-19.

We do not know how the experimental vaccine may affect a fetus or nursing infant. Therefore, women who can become pregnant must have a negative pregnancy test before each injection and must agree to use effective birth control beginning at least 21 days before the first vaccination until the end of the study.

The remaining document will now describe the research study in more detail. This information should be considered before you make your choice. Members of the study team will talk with you about the information in this document. Some people have personal, religious, or ethical beliefs that may limit the kinds of medical or research interventions in which they would want to participate. Take the time you need to ask any questions and discuss this study with NIH staff, and with your family, friends, and personal health care providers.

IT IS YOUR CHOICE TO TAKE PART IN THE STUDY

You may choose not to take part in this study for any reason. If you join this study, you may change your mind and stop participating in the study at any time and for any reason. In either case, you will not lose any benefits to which you are otherwise entitled. However, to be seen at the NIH, you must be taking part in a study or are being considered for a study. If you do choose to leave the study, please inform your study team to ensure a safe withdrawal from the research.

WHY IS THIS STUDY BEING DONE?

Nipah virus is transmitted from animals to humans, from human to human and through contaminated food. In infected people, the virus causes mild to severe illness. Some people have no symptoms, some may have respiratory illness, such as cough and difficulty breathing. People with severe symptoms may have brain swelling which can lead to death. Although Nipah virus has caused only a few known outbreaks in Asia, it infects a wide range of animals and can cause severe disease and death in people, making it a public health concern. There are currently no drugs or vaccines to treat or prevent Nipah virus infection.

This is a study of an experimental vaccine called mRNA-1215 for prevention of the Nipah virus (NiV) infection. The experimental vaccine is a messenger ribonucleic acid (mRNA) vaccine made by Moderna. This study vaccine is made of the genetic code of the virus. This genetic code will make a protein from the virus and cause your body to think you have been infected with the virus. It is not made from the Nipah virus and cannot cause Nipah virus infection. This study vaccine has not been given to humans before this study.

The main purpose of this study is to see if the experimental vaccine is safe and how your body responds to it. Since this is the first time we are giving mRNA-1215 to people, we do not know how your body will respond. This study is not designed to protect you from Nipah virus. We will tell you if we learn anything new during this study that might cause you to change your mind about staying in the study. At the end of the study, we will tell you when study results may be available and how to learn about them.

We are asking you to join this research study because you are a healthy adult and qualify for participation based on screening evaluations.

The mRNA-1215 vaccine is an investigational product, which means that it has not been approved by the U.S. Food and Drug Administration (FDA) to use for prevention of NiV infection. The FDA allows this experimental product to be used for research only.

WHAT WILL HAPPEN DURING THE STUDY?

The table below shows the plan for this study.

Study Plan				
**Group**	**Number of Participants**	**Dose/Route**	**Day 0**	**Month 1**
1	10	25 mcg IM	X	X
2	10	50 mcg IM	X	X
3	10	100 mcg IM	X	X
4	10	10 mcg IM	X	X
**Total**	40			

If you decide to take part in this study, you will be asked to review and agree to this informed consent form and the procedures it describes. You will have completed the screening process

which includes a physical exam and review of your medical history, vital signs and laboratory results, including an HIV test. You must be healthy and qualify for enrollment before you can take part in this study.

The study opened with enrollments into Group 1 for the 25 mcg mRNA-1215 dose. After the study team agreed that there were no safety concerns with the vaccine, Group 2 and Group 3 opened. The dose for Group 4 was selected based on the data from Groups 1-3. At this time, all new enrollments will be assigned to Group 4 because Groups 1-3 have already been filled.

If you are female and able to become pregnant, you must use an effective method of birth control for the entire study, beginning 21 days prior to enrollment. You will be given a pregnancy test before each vaccination. If you are pregnant, we will not give you mRNA-1215.

Vaccine Administration

The injection (shot) will be given in the upper arm muscle. This is called an intramuscular "IM" injection. We will use a needle and syringe to give you the injection.

You will need to stay in the clinic for at least 30 minutes after each vaccination. You may need to stay longer than 30 minutes if you feel unwell or have symptoms. In keeping with the NIH CC policy and good medical practice, medical care will be given to subjects for any immediate allergic reactions or other injury resulting from participation in this research study.

Follow-Up after Vaccination

The day after your vaccination, clinic staff will call to check on you. Also, after each vaccination, you will need to complete a diary card. You will need to record any symptoms that you may have as a result of the vaccination on the diary card for 7 days. We will give you a thermometer to check your temperature every day. You will need to check your temperature and complete your diary card even if you feel well and have no symptoms. We will also give you a ruler to measure any skin changes at the injection site. You will get a password to a secure website where you can enter this data online. If you do not have access to the internet, you can use a paper diary card instead.

If you have any symptoms or feel unwell at any time during the study, you should tell a clinic nurse or doctor as soon as possible. You can reach the staff by phone 24 hours a day. If you have symptoms, you may be asked to come to the clinic for a checkup. It is very important that you follow the instructions from the clinic staff.

You will have several follow-up visits during this study. These visits allow us to check you for any health changes or problems. We will ask you how you are feeling and if you have taken any medications.

We will draw about 3 to 14 tubes or 28 to 136 mL (about 2 to 9 tablespoons) of blood at each follow-up visit. In keeping with NIH Clinical Center guidelines, we will not draw more than 550 mL of blood from you in any 8-week period. Some of the blood that we take will be used for research and some will be used to monitor your health. We will tell you right away if any of the tests done to monitor your health show a problem. You might need to have extra clinic visits or laboratory tests if you have health changes that need to be checked.

Clinical studies follow a set schedule. It is important that you follow the schedule as closely as possible. You should try to not miss any visits. You should contact the clinic staff as soon as possible if you need to change the date or time of any study visit.

Genetic Testing

Some of the blood drawn from you for research purposes will be used for genetic tests. Some genetic tests are done in research studies to see if there are genetic differences in immune responses. Your blood samples used in these genetic tests will not have your name on it, and the results will not be in your medical record. You will not get results of these tests.

Apheresis

We would like to collect your blood one time by a method called "apheresis" at 2 weeks after your second vaccination. This procedure is optional and choosing not to take part will not affect your study participation. To be eligible for apheresis, you must not:

- have an unstable heart as indicated by your medical history and test results
- have blood pressure greater than 180/100
- have a known blood clotting disorder
- be pregnant or breast feeding
- have a condition that the attending physician or the apheresis clinic staff considers a reason to not do an apheresis procedure.

Before apheresis, we will check your weight, pulse and blood pressure. We will ask questions about your general health and medical history. If you are female and able to become pregnant, we will do a pregnancy test before the apheresis procedure. The test must show that you are not pregnant. The apheresis staff will prick your finger to test your blood for anemia before the procedure.

During the procedure, you will lay on a reclining chair or hospital bed. A sterile needle will be placed into a vein in both of your arms. The kits used to collect apheresis samples are sterile, single-use, disposable sets that are not in contact with any person's bodily fluids other than yours. No blood products are given to you during the procedure. Apheresis is done at the NIH Clinical Center, and a physician from the NIH Department of Transfusion Medicine will be available in or near the apheresis area at all times.

In the apheresis procedure, blood is removed through a needle in the vein of one arm, spun in a machine that separates the white blood cells and then the rest of your blood is returned to you through a needle in the other arm. A medication called Citrate is added to the blood while in the machine to prevent your blood from clotting.

The purpose of this procedure is to allow us to get a large number of white blood cells that cannot be collected by simple blood drawing. We want to study these white blood cells. The number of white blood cells collected is a small fraction of the total amount in your body. The body quickly replaces the cells that have been removed. The NIH Blood Bank at the Clinical Center and other blood banks use similar procedures every day to collect blood samples from donors. We will not use your samples for transfusion or therapy.

HOW LONG WILL THE STUDY TAKE?

If you agree to take part in this study, your involvement is expected to last for 52 weeks (1 year) after your last vaccination.

Participants in all groups will have a total of 2 vaccination visits, 11 follow-up clinic visits, and 2 follow-up phone calls over 56 weeks. Each vaccination visit will take about 6 hours. Most other follow-up clinic visits will take about 1 to 2 hours. The optional apheresis visit will take 3 to 4 hours.

HOW MANY PEOPLE WILL PARTICIPATE IN THIS STUDY?

We plan to enroll about 40 people in this study. This includes 10 people in the 25 mcg dose group, 10 people who in the 50 mcg dose group, 10 people in the 100 mcg dose group, and 10 people who will get the 10 mcg dose.

WHAT ARE THE RISKS AND DISCOMFORTS OF BEING IN THE STUDY?

Possible Risks of the mRNA-1215 vaccine

The mRNA-1215 vaccine has not been given to people before this study. It may have unknown risks. It has been tested in animals. The vaccine did not cause any unusual side effects in animals and met the safety criteria to be tested in humans.

You may experience any of the following: feeling tired/unwell, fever, muscle aches, headache, chills, nausea, joint pain, or injection site reactions including pain/tenderness, redness, swelling, itchiness at the injection site, and lymph node swelling. These side effects also commonly occur with FDA approved vaccines. The side effects usually occur within the first 24 hours after the vaccine is given and go away within a few days to a week.

Rarely, a serious allergic reaction with symptoms like hives, rash, sweating, swelling around the mouth, throat or eyes, wheezing, trouble breathing, increased pulse or sudden weakness may occur shortly after any vaccination. This is called “anaphylaxis” and may be life threatening. While you are waiting in the clinic after vaccination, we will monitor you for anaphylaxis. Treatment for anaphylaxis will be given right away if it occurs. Also rarely, hives may occur after any vaccination and may last for days to several months. If you have hives, we will treat you and monitor you frequently.

Recently, rare cases of myocarditis (inflammation of the heart muscle) and pericarditis (inflammation of the lining around the heart) have been reported in males and females after they received the COVID-19 mRNA vaccines. Most of the cases of myocarditis or pericarditis happened in males and females shortly after the second dose of the vaccine. The risk was highest in males under the age of 40, specifically males between 12 and 17 years old.

Symptoms of myocarditis or pericarditis include:

- Chest pain
- Shortness of breath
- Feelings of having a fast-beating, fluttering, or pounding heart

These symptoms start most commonly within a few days after study vaccination. You should seek medical attention and also notify study staff if you have any of these symptoms. Although some people who had myocarditis or pericarditis had a severe case, most people felt better quickly. Long term follow-up information on these rare situations is limited at this time. We do not know if the risk of myocarditis or pericarditis may be increased following additional doses of the vaccine.

Possible Risks of Vaccine Injections

Temporary stinging, pain, redness, soreness, itchiness, swelling, or bruising may occur at the site of the injection.

Possible Risks of Blood Drawing

Blood drawing may cause pain, bruising, and lightheadedness or fainting. Very rarely, an infection at the site where the blood is taken may occur.

Possible Risks of Apheresis

Apheresis is generally safe, and side effects are rare. Pain, bruising or discomfort at the needle placement site may occur. Sometimes apheresis causes a tingling sensation around the lips, nose and mouth, feeling chilled, and/or slight nausea. This can usually be relieved by slowing or temporarily stopping the apheresis or taking a calcium pill, like Tums®. Other possible side effects are anxiety, vomiting, and lightheadedness. Temporary lowering of the blood pressure may develop. There is the rare possibility of infection, fainting, or seizure. Very rarely a nerve problem at the needle placement site may occur. Also, very rarely, a machine malfunction may occur and result in the loss of about one unit (one pint) of blood.

There are theoretical risks from re-infusion of the blood after processing by the machine such as infection or an adverse reaction to the blood components. However, this has not been seen in many thousands of volunteers who have undergone this or similar procedures to date. There may be other risks of apheresis that are unknown at this time.

During the procedure, your platelet count may decrease because platelets are collected with the white blood cells. Platelets are cells that help your blood to clot. Taking aspirin in combination with a lowered platelet count may increase your chance of developing bleeding. Therefore, you should not take aspirin or aspirin-containing drugs for 2 weeks after the procedure without physician approval.

Unknown Safety Risks

There may be side effects from the study vaccine - even serious or life-threatening ones - that we do not yet know about. Please tell the study staff about any side effect you think you are having. This is important for your safety.

Possible Risks from Stored Samples

We will collect blood samples from you during the study. We will keep these samples indefinitely for future research to learn more about flu virus, vaccines, the immune system, and other research questions. Results from research with your samples will not be in your medical record or reported to you.

Labeling of Stored Samples: Your stored samples will be labeled by a special code or number and not your personal information. Only the study team can link this code to you. Any identifying information about you (like name or date of birth) will be kept as confidential as allowed by law.

Risks of Stored Samples: There is a small chance that information from your medical records could be given to someone who should not get it without your permission. It is possible for someone to use that information to discriminate against you when you apply for insurance or employment. Similar problems may occur if you give information about yourself or agree to have your medical records released.

Possible Risks of Data Sharing

Information in the shared databases could be linked back to you and used to discriminate against you or your family. State and federal laws provide some protections against genetic and preexisting conditions discrimination.

Possible Other Risks

We do not know if the study vaccine will change how your body responds to Nipah virus infections in the future. You may not donate blood at a blood bank while taking part in this study. You may not donate blood for one year after the last experimental vaccine injection.

What are the risks related to pregnancy?

We do not know how the experimental vaccine may affect a fetus or nursing infant. Therefore, women who are able to become pregnant must have a negative pregnancy test before each vaccine injection and must agree to use effective birth control beginning at least 21 days before the first injection until the end of the study. We will discuss effective methods of birth control with you. Any time during the study you must tell the clinic staff right away if you become pregnant, your birth control method fails, or you think that you might be pregnant. If you are pregnant, you will be asked to continue with follow-up visits so that we can check your health. We will contact you to ask about the outcome of the pregnancy.

WHAT ARE THE BENEFITS OF BEING IN THE STUDY?

You will not benefit from being in this study.

Are there any potential benefits to others that might result from the study?

In the future, other people might benefit from this study because of knowledge we gain about vaccines and immune responses against Nipah virus.

WHAT OTHER OPTIONS ARE THERE FOR YOU?

Before you decide whether or not to be in this study, we will discuss other options that are available to you. Instead of being in this study, you could choose not to take part. You may be eligible for other VRC studies.

DISCUSSION OF FINDINGS

New information about the study

If we find out any new information that may affect your choice to participate in this study, we will get in touch with you to explain what we have learned. This may be information we have learned while doing this study here at the NIH or information we have learned from other scientists doing similar research in other places.

Return of research results

At each visit you will be checked for any health changes or problems. Blood will be drawn at almost every study visit to check on your health. You will be informed right away if any of your test results show a health problem. The results of this study may be reported in medical journals, on the internet or at scientific meetings. We will give you information about how to find the study results once they are available.

EARLY WITHDRAWAL FROM THE STUDY

Your participation in this study is completely voluntary. You can choose to stop taking part in the study at any time. There is no penalty or loss of benefits if you choose to leave the study. However, if you get at least one dose of mRNA-1215 during this study, we encourage you to take part in safety follow-up. It is important that we continue to check your health.

You may be removed from the research study for any of the following reasons:

- You don't keep appointments or follow study procedures;
- You get a serious illness that needs ongoing medical care;
- The researcher believes that it is in your best interest to remove you from the study;
- The study is stopped by regulatory agencies, the study sponsor, or study investigators. If this happens, we will tell you why.

The study team may also decide to keep you in the study but to stop giving you study product. If you need a treatment with a medication that affects your immune system (such as a steroid like prednisone), please inform the study team as soon as possible. In this case, the second vaccine administration may be discontinued or delayed up to 2 weeks after the treatment.

If you are not able to get the second injection or it is discontinued by the researchers, we may adjust your follow-up schedule but will ask that you still attend study visits.

STORAGE, SHARING AND FUTURE RESEARCH USING YOUR SPECIMENS AND DATA

Will your specimens or data be saved for use in other research studies?

As part of this study, we are obtaining specimens and data from you. We will remove all the identifiers, such as your name, date of birth, address, or medical record number and label your specimens and data with a code so that you cannot easily be identified. However, the code will be linked through a key to information that can identify you. We plan to store and use these specimens and data for studies other than the ones described in this consent form that are going on right now, as well as studies that may be conducted in the future. These studies may provide

additional information that will be helpful in understanding Nipah virus, or other diseases or conditions. This could include studies to develop research tests, treatments, drugs, or devices, that may lead to the development of a commercial product by the NIH and/or its research or commercial partners. You will not be provided financial compensation if this happens. Also, it is unlikely that we will learn anything from these studies that may directly benefit you.

If you agree to take part in this study, you give permission for your coded specimens and data to be stored and used for future research as described above.

Will your specimens or data be shared for use in other future research studies?

We may share your coded specimens and data with other researchers. If we do, while we will maintain the code key, we will not share it, so the other researchers will not be able to identify you. They may be doing research in areas that are similar to this study or in other unrelated areas. These researchers may be at NIH, other research centers and institutions, or commercial entities.

By agreeing to take part in this study, you give permission for your coded specimens and data to be shared with other researchers and used by these researchers for future research as described above.

If you change your mind and do not want us to use your specimens and data for future research, you should contact the research team member identified at the top of this document. We will do our best to comply with your request but cannot guarantee it. For example, if research with your specimens and/or data has already been completed, the information from that research may still be used. Also, for example, if the specimens and data have been shared already with other researchers, it might not be possible to withdraw them.

In addition to the planned use and sharing described above, we might remove all identifiers and codes from your specimens and data and use or share them with other researchers for future research at the NIH or other places. When we or the other researchers access your anonymized data, there will be no way to link the specimens or data back to you. We will not contact you to ask your permission or otherwise inform you before we do this. If we do this, we would not be able to remove your specimens or data to prevent their use in future research studies, even if you asked, because we will not be able to tell which are your specimens or data.

NIH policies require that your clinical and other study data be placed in an internal NIH database that is accessible to other NIH researchers for future research. Usually, these researchers will not have access to any of your identifiers, such as your name, date of birth, address, or medical record number; and your data will be labeled with only a code. We cannot offer you a choice of whether your data to be placed in this database or not. If you do not wish to have your data placed in this database, you should not enroll in this study.

If you decide not to take part in this study, you may still take part in other studies at NIH.

How long will your specimens and data be stored by the NIH?

Your specimens and data may be stored by the NIH indefinitely.

Risks of storage and sharing of specimens and data

When we store your specimens and data, we take precautions to protect your information from others that should not have access to it. When we share your specimens and data, we will do everything we can to protect your identity, for example, when appropriate, we remove information that can identify you. Even with the safeguards we put in place, we cannot guarantee that your identity will never become known, or someone may gain unauthorized access to your information. New methods may be created in the future that could make it possible to re-identify your specimens and data.

PAYMENT

Will you receive any type of payment for taking part in this study?

Some NIH Clinical Center studies offer compensation for participation in research. The amount of compensation, if any, is guided by NIH policies and guidelines. You will be compensated for your time and inconvenience by the NIH Clinical Research Volunteer Program. It is possible that you may have some expenses that are not covered by the compensation provided.

The compensation is as follows:

- $315 per vaccination visit
- $25 total for the timely completion of all 7 days of an electronic diary
- $200 for each scheduled follow-up visit that includes a research blood draw
- $85 for all other clinic visits that do not include research blood draws
- $285 for optional apheresis

The total compensation you get is based on the number and type of study visits you complete. You will get the compensation about 2 weeks after each completed visit. You may choose to receive payments by direct deposit into a bank account or to an actual or digital debit card. If you are unable to finish the study, you will receive compensation only for the parts and visits you completed.

To receive compensation, you will be asked to provide your social security number. You can withhold your social security number and still participate in research, but you may not be able to receive compensation. With few exceptions, the NIH is required to report all payments given to research participants to the Internal Revenue Service (IRS). Study compensation is considered taxable income that is reportable to the Internal Revenue Service (IRS). A "Form 1099-Other Income" will be sent to you if your total payments for research participation are $600 or more in a calendar year. Payments are processed through the U.S. Treasury. If you have unpaid debt to the federal government, the U.S. Treasury may deduct all or some of your compensation. They will notify you directly if this happens.

REIMBURSEMENT

Will you receive reimbursement or direct payment by NIH as part of your participation?

This study does not offer reimbursement for participants, or payment of, hotel, travel, or meals.

COSTS

Will taking part in this research study cost you anything?

NIH does not bill health insurance companies or participants for any research or related clinical care that you receive at the NIH Clinical Center.

There are no costs to you for participating in this study. You or your health insurance will have to pay for all medical costs for medical care that you get outside this study. It is possible that you may have some expenses that are not covered by the study compensation provided.

CONFLICT OF INTEREST (COI)

The NIH reviews NIH staff researchers at least yearly for conflicts of interest. This process is detailed in a COI Guide. You may ask your research team for a copy of the COI Guide or for more information. Members of the research team who do not work for NIH are expected to follow these guidelines or the guidelines of their home institution, but they do not need to report their personal finances to the NIH.

Moderna is providing mRNA-1215 for this study to NIH without charge. No NIH investigator involved in this study receives payments or other benefits from any company whose drug, product or device is being tested. However, there are some research partners not associated with the NIH working on this study who may receive payments or benefits, limited by the rules of their workplace.

CLINICAL TRIAL REGISTRATION AND RESULTS REPORTING

A description of this clinical trial will be available on www.ClinicalTrials.gov as required by U.S. Law. This website will not include information that can identify you. At most, the website will include a summary of the results. You can search this Web site at any time.

CONFIDENTIALITY PROTECTIONS PROVIDED IN THIS STUDY

Will your medical information be kept private?

We will do our best to make sure that the personal information in your medical record will be kept private. However, we cannot guarantee total privacy. Organizations that may look at and/or copy your medical records for research, quality assurance, and data analysis include:

- The NIH and other government agencies, like the Food and Drug Administration (FDA), which are involved in keeping research safe for people.
- National Institutes of Health Intramural Institutional Review Board
- The study Sponsor (DMID) or their agent(s)
- Qualified representatives from Moderna, the pharmaceutical company who produces mRNA-1215.

The researchers conducting this study and the NIH follow applicable laws and policies to keep your identifying information private to the extent possible. However, there is always a chance that, despite our best efforts, your identity and/or information about your participation in this research may be inadvertently released or improperly accessed by unauthorized persons.

In most cases, the NIH will not release any identifiable information collected about you without your written permission. However, your information may be shared as described in the section of this document on sharing of specimens and data, and as further outlined in the following sections.

Further, the information collected for this study is protected by NIH under a Certificate of Confidentiality and the Privacy Act.

Certificate of Confidentiality

To help us protect your privacy, the NIH Intramural Program has received a Certificate of Confidentiality (Certificate). With this certificate, researchers may not release or use data or information about you except in certain circumstances.

NIH researchers must not share information that may identify you in any federal, state, or local civil, criminal, administrative, legislative, or other proceedings, for example, if requested by a court.

The Certificate does not protect your information when it:

1. is disclosed to people connected with the research, for example, information may be used for auditing or program evaluation internally by the NIH; or
2. is required to be disclosed by Federal, State, or local laws, for example, when information must be disclosed to meet the legal requirements of the federal Food and Drug Administration (FDA);
3. is for other research;
4. is disclosed with your consent.

The Certificate does not prevent you from voluntarily releasing information about yourself or your involvement in this research.

The Certificate will not be used to prevent disclosure to state or local authorities of harm to self or others including, for example, child abuse and neglect, and by signing below you consent to those disclosures. Other permissions for release may be made by signing NIH forms, such as the Notice and Acknowledgement of Information Practices consent.

Privacy Act

The Federal Privacy Act generally protects the confidentiality of your NIH medical information that we collect under the authority of the Public Health Service Act. In some cases, the Privacy Act protections differ from the Certificate of Confidentiality. For example, sometimes the Privacy Act allows release of information from your record without your permission, for example, if it is requested by Congress. Information may also be released for certain research purposes with due consideration and protection, to those engaged by the agency for research purposes, to certain federal and state agencies, for HIV partner notification, for infectious disease or abuse or neglect reporting, to tumor registries, for quality assessment and medical audits, or when the NIH is involved in a lawsuit. However, NIH will only release information from your medical record if it is permitted by both the Certificate of Confidentiality and the Privacy Act.

POLICY REGARDING RESEARCH-RELATED INJURIES

The NIH Clinical Center will provide short-term medical care for any injury resulting from your participation in research here. In general, no long-term medical care or financial compensation for research-related injuries will be provided by the NIH, the NIH Clinical Center, or the Federal Government. However, you have the right to pursue legal remedy if you believe that your injury justifies such action.

PROBLEMS OR QUESTIONS

If you have any problems or questions about this study, or about your rights as a research participant, or about any research-related injury, contact the Principal Investigator, Lesia Dropulic, MD, [redacted] Other researchers you may call are: Laura Novik [redacted]5. You may also call the NIH Clinical Center Patient Representative at 301-496-2626, or the NIH Office of IRB Operations at 301-402-3713, if you have a research-related complaint or concern.

CONSENT DOCUMENT

Please keep a copy of this document in case you want to read it again.

Adult Research Participant: I have read the explanation about this study and have been given the opportunity to discuss it and to ask questions. I consent to participate in this study.

Signature of Research Participant	Print Name of Research Participant	Date

Investigator:

Signature of Investigator	Print Name of Investigator	Date

Witness should sign below if either:

1. **A short form consent process has been used to enroll a non-English speaking subject or**
2. **An oral presentation of the full consent has been used to enroll a blind or illiterate subject**

Signature of Witness	Print Name of Witness	Date

NIH ADMINISTRATIVE SECTION TO BE COMPLETED REGARDING THE USE OF AN INTERPRETER:

____ An interpreter, or other individual, who speaks English and the participant's preferred language facilitated the administration of informed consent and served as a witness. The investigator obtaining consent may not also serve as the witness.

____ An interpreter, or other individual, who speaks English and the participant's preferred language facilitated the administration of informed consent but did not serve as a witness. The name or ID code of the person providing interpretive support is: ______________________________.
