# Supplementary material for: A structure-based mRNA vaccine for Nipah virus in healthy adults: a phase 1 trial
Source: Nat Med. 2026 Mar 12;32(4):1401–10. doi: 10.1038/s41591-026-04265-1 (PMC13099371; doi:10.1038/s41591-026-04265-1)
Supplement: Supplementary file 2 — Reporting Summary [file 41591_2026_4265_MOESM2_ESM.pdf]

Corresponding author(s): Lesia K. Dropulic

Last updated by author(s): 12/18/2025

Reporting Summary

Nature Portfolio wishes to improve the reproducibility of the work that we publish. This form provides structure for consistency and transparency in reporting. For further information on Nature Portfolio policies, see our Editorial Policies and the Editorial Policy Checklist.

Statistics

For all statistical analyses, confirm that the following items are present in the figure legend, table legend, main text, or Methods section.

n/a	Confirmed	
☐	☒	The exact sample size (*n*) for each experimental group/condition, given as a discrete number and unit of measurement
☐	☒	A statement on whether measurements were taken from distinct samples or whether the same sample was measured repeatedly
☐	☒	The statistical test(s) used AND whether they are one- or two-sided<br>*Only common tests should be described solely by name; describe more complex techniques in the Methods section.*
☐	☒	A description of all covariates tested
☐	☒	A description of any assumptions or corrections, such as tests of normality and adjustment for multiple comparisons
☐	☒	A full description of the statistical parameters including central tendency (e.g. means) or other basic estimates (e.g. regression coefficient) AND variation (e.g. standard deviation) or associated estimates of uncertainty (e.g. confidence intervals)
☐	☒	For null hypothesis testing, the test statistic (e.g. *F*, *t*, *r*) with confidence intervals, effect sizes, degrees of freedom and *P* value noted<br>*Give P values as exact values whenever suitable.*
☒	☐	For Bayesian analysis, information on the choice of priors and Markov chain Monte Carlo settings
☒	☐	For hierarchical and complex designs, identification of the appropriate level for tests and full reporting of outcomes
☐	☒	Estimates of effect sizes (e.g. Cohen's *d*, Pearson's *r*), indicating how they were calculated

Our web collection on statistics for biologists contains articles on many of the points above.

Software and code

Policy information about availability of computer code

Data collection	ELISA data were collected using an Envision plate reader (PerkinElmer); neutralization activity was measured using SpectraMax L luminometer (Molecular Devices); B and T cell data were acquired using BD FACSymphony cytometer; Clinical data : AdvantageEDC (SM), Regulatory Tracking System (RTS) maintained and hosted by by the Clinical Program Support Center (CPSC) at the EMMES Corporation.
Data analysis	R version 4.3.3., mmrm package in R; Prism, versions 10.0.0, 10.2.2, and 10.4.1, FlowJo version 10.10.0.

For manuscripts utilizing custom algorithms or software that are central to the research but not yet described in published literature, software must be made available to editors and reviewers. We strongly encourage code deposition in a community repository (e.g. GitHub). See the Nature Portfolio guidelines for submitting code & software for further information.

Data

Policy information about availability of data

All manuscripts must include a data availability statement. This statement should provide the following information, where applicable:

- Accession codes, unique identifiers, or web links for publicly available datasets
- A description of any restrictions on data availability
- For clinical datasets or third party data, please ensure that the statement adheres to our policy

Results generated in this study are available as de-identified data on ClinicalTrials.gov. https://clinicaltrials.gov/study/NCT05398796?cond=Nipah&term=vaccine&rank=2&a=51&b=52. The study protocol and informed consent form are available on ClinicalTrials.gov (https://clinicaltrials.gov/study/

NCT05398796?term=VRC%20322&rank=1) Additional data, including de-identified individual level data, may be made available upon reasonable request to the corresponding author sent to dropulic@niaid.nih.gov for investigators whose proposed use of the data has been approved by the National Institutes of Health (NIH) Institutional Review Board. The anticipated timeframe for a response to such a request is about 1 to 3 weeks. A signed data access agreement is required before data sharing. Access to data is dependent on the time to establish a signed agreement between the NIH and the requesting investigator. This may take up to 1 to 3 months. Access to data is usually not time limited after a signed agreement is executed and data is transferred.

Research involving human participants, their data, or biological material

Policy information about studies with human participants or human data. See also policy information about sex, gender (identity/presentation), and sexual orientation and race, ethnicity and racism.

Reporting on sex and gender	Participants self-reported sex on the day of the enrollment and these results are reported in Supplementary Table 1. No sex or gender based analyses were performed as this was not an objective of the trial. Such investigation is beyond the scope of these small phase 1 clinical trials and should be further investigated in the subsequent clinical investigation phases.
Reporting on race, ethnicity, or other socially relevant groupings	Participants self-reported on their race and ethnicity. Participants chose their race from options including Asian, Black or African American, White, and Multiracial and their ethnicity as either Non-Hispanic or Latino or Hispanic/Latino. For both race and ethnicity, it was permitted to decline self-reporting of these categories, in which case participants are reported as Unknown/Not reported.
Population characteristics	Eligible participants were adults 18 to 60 years of age who were in good general health, as determined by medical history, physical examination, and laboratory testing. Exclusion criteria related to the pathogen target of the mRNA-1215 vaccine were confirmed past Nipah virus infection or prior residence for greater than 6 months or planned travel during the study for any length of time to places where Nipah virus infection is endemic.<br>Forty participants, 18 females (45%) and 22 males (55%), with an overall mean age of 37 (range 22 to 59) were enrolled into the study from July 11, 2022 to August 22, 2023
Recruitment	Participants were recruited from the greater Washington, D.C. area using IRB-approved recruitment ads. the trial was open-labeled and non-randomized, which could potentially introduce an element of bias in the reporting of reactogenicity.
Ethics oversight	This phase I clinical trial was reviewed and approved by the NIH Institutional Review Board (IRB).

Note that full information on the approval of the study protocol must also be provided in the manuscript.

Field-specific reporting

Please select the one below that is the best fit for your research. If you are not sure, read the appropriate sections before making your selection.

☒ Life sciences ☐ Behavioural & social sciences ☐ Ecological, evolutionary & environmental sciences

For a reference copy of the document with all sections, see nature.com/documents/nr-reporting-summary-flat.pdf

Life sciences study design

All studies must disclose on these points even when the disclosure is negative.

Sample size	Sample size determination for this trial was based on the primary endpoint of safety. These calculations for safety were predetermined and expressed in terms of the ability to detect SAEs. Sample sizes were chosen so that there was a 90% chance to observe at least one SAE if the true rate was at least 0.21, and over a 90% chance to observe no SAE if the true rate was no more than 0.01. The study was not designed to detect large immunologic differences between the groups (i.e. 1.2 times the standard deviation of the immune response with 80% power). Adjustments for multiple comparisons were not performed.
Data exclusions	One participant in the 10 mcg group received only a single dose of mRNA-1215 and data collected only at weeks 0, 2, and 4 was included in the analysis for this participant. No other data was excluded.
Replication	Each ELISA assay was done in triplicate with duplicate values inside each assay. A plasma positive control present on each plate was used to determine if the assay passed or failed according to acceptance criteria pre-established in advance; only passed assays were analyzed and reported. Five plasma bridge controls, included for each assay, were also used to assess reproducibility across assays. For measured samples, from the triplicate assays, any sample with a CV>30% across the 3 EC50 values determined was rerun in a 4th assay and that 4th value was included in the final analysis.Neutralization assays were run in duplicate or triplicate with control antibody samples included in each plate for reproducibility.
Randomization	This trial was non-randomized. The study group assignment was set up in the database prior to opening the study to accrual. The group assignment was known to the staff and study participants before completing the electronic enrollment into the study on Day 0. The assignment to a group was not randomized because this is not crucial for descriptive and exploratory research that is not evaluating a treatment effect as would occur in a phase 2 or 3 study.
Blinding	We did not include blinding in this trial, because it was not needed to assess safety and tolerability or immunogenicity in this first-in-human trial of a novel vaccine administered to a naïve population.

Reporting for specific materials, systems and methods

We require information from authors about some types of materials, experimental systems and methods used in many studies. Here, indicate whether each material, system or method listed is relevant to your study. If you are not sure if a list item applies to your research, read the appropriate section before selecting a response.

Materials & experimental systems

n/a	Involved in the study
☐	☒ Antibodies
☐	☒ Eukaryotic cell lines
☒	☐ Palaeontology and archaeology
☒	☐ Animals and other organisms
☐	☒ Clinical data
☒	☐ Dual use research of concern
☒	☐ Plants

Methods

n/a	Involved in the study
☒	☐ ChIP-seq
☐	☒ Flow cytometry
☒	☐ MRI-based neuroimaging

Antibodies

Antibodies used

ELISA: anti-non-human primate IgG-horseradish peroxidase conjugate cross-reacting to human IgG, Southern Biotech Catalogue number #2040-05-Lot E0922-Z742C expiration date 2024-06; Lot C3923-XJ83C expiration date 2025-07

T cell analysis:
1. Live/dead fixable aqua dead cell stain, Invitrogen #L34957 – Lot #2696857[1:800]
2. CD45RA PE-CY5, clone 5H9, BD Biosciences #552888 – Lot #1118897[1:2500]
3. CD4 PE-CY5.5, clone SK3, Thermo Fisher #35-0047-42 – Lot #2521213[1:2500]
4. ICOS PE-CY7, clone C398.4A, Biolegend #313520 – Lot #B293719[1:640]
5. CD8 BV570, clone RPA-T8, Biolegend #301038 – Lot #B367805[1:80]
6. CCR7 BV650, clone GO43H7, Biolegend #353234 – Lot #B370728[1:10]
7. CXCR3 BV711, clone 1C6/CXCR3, BD Biosciences #563156 – Lot #2129036[1:20]
8. PD-1 BUV737, clone EH12.1, BD Horizon #612792 – Lot #3220435[1:20]
9. TNF FITC, clone Mab11, BD Biosciences #554512 – Lot #2213980[1:80]
10. IL-4 BB700, clone MP4-25D2, BD Biosciences custom order – Lot #1145122[1:20]
11. CXCR5 PE, clone MU5UBEE, Thermo Fisher #12-9185-42 – Lot #2404260[1:10]
12. CD69 ECD, clone TP1.55.3, Beckman Coulter #7620104 [1:40]
13. IL-21 Ax647, clone 3A3-N2.1, BD Biosciences #560493 – Lot #2266971[1:10]
14. IFN-g Ax700, clone B27, Biolegend #506516 – Lot #B320892[1:640]
15. CD3 APC-CY7, clone SP34.2, BD Biosciences #557757 – Lot #1152687[1:320]
16. IL-13 BV421, clone JES10-5A2, BD Biosciences #563580 – Lot #3086969[1:20]
17. IL-17A BV605, clone BL168, Biolegend #512326 – Lot #B376461[1:20]
18. CD154 BV785, clone 24-31, Biolegend #310842 – Lot #B329207[1:20]
19. IL-2 BV750, clone MQ1-17H12, BD Biosciences #566361 – Lot #2285235[1:40]

B cell analysis:
1. Live/dead fixable aqua dead cell stain, Invitrogen #L34957 – Lot #2696857[1:800]
2. IgD FITC, goat pAb, Southern Biotech #2030-02 – Lot #A2118-WF09C [1:160]
3. IgM PerCP-Cy5.5, clone G20-127, BD Biosciences #561285 – Lot #0307134[1:40]
4. IgA Dy405, goat pAb, Jackson ImmunoResearch #109-475-011 – Lot #150866 [1:80]
5. CD20 BV570, clone 2H7, Biolegend #302332 – Lot #B301458[1:40]
6. CD27 BV650, clone O323, Biolegend #302828 – Lot #B350350[1:20]
7. CD14 BV785, clone M5E2, Biolegend #301840 – Lot #B327948 [1:80]
8. CD16 BUV496, clone 3G8, BD Biosciences #564653 – Lot #0155949[1:80]
9. CD4 BUV737, clone SK3, BD Biosciences #564305 – Lot #0282762[1:320]
10. CD19 APC, clone J3-119, Beckman Coulter #IM2470U – Lot #200093[1:80]
11. IgG Ax700, clone G18-145, BD Biosciences #561296 – Lot #0135021[1:20]
12. CD3 APC-Cy7, clone SP34.2, BD Biosciences #557757 – Lot #1152687[1:40]
13. CD38 PE, clone OKT10, Caprico Biotech #100826 – Lot #8AE4[1:160]
14. CD21 PE-Cy5, clone B-ly4, BD Biosciences #551064 – Lot #0072939[1:40]
15. CXCR5 PE-Cy7, clone MU5UBEE, Thermo Fisher #25-9185-42 – Lot #2442267[1:40]

Validation

All antibodies were commercially manufactured and validated. ELISA: Each lot of antibody was thoroughly bridged to match historical potency and reproducibility with a series of plasma samples from both non-human primate and human origin. In such the dilution at which each antibody was used differed from lot-to-lot. All antibodies for flow cytometry were titrated on human PBMC and the concentrations that gave the best separation were selected. Representative staining is shown in the gating trees for the Intracellular Cytokine Staining and B cell panels (Extended Data Figure 2,3).

Eukaryotic cell lines

Policy information about cell lines and Sex and Gender in Research

Cell line source(s)	HEK293T (ATCC #CRL-11268) is a cell line exhibiting epithelial morphology that was isolated from human embryo kidney tissue.
Authentication	Since the HEK293Tcell line was sourced from ATCC, independent authentication was not done. ATCC characterization report can be found here https://www.atcc.org/products/crl-11268
Mycoplasma contamination	Cell lines were not tested for mycoplasma contamination.
Commonly misidentified lines (See ICLAC register)	None

Clinical data

Policy information about clinical studies

All manuscripts should comply with the ICMJE guidelines for publication of clinical research and a completed CONSORT checklist must be included with all submissions.

Clinical trial registration	NCT05398796
Study protocol	The protocol is available in ClinicalTrials.gov under the corresponding trial registration number, as well as in the Supplementary Appendix for this manuscript.
Data collection	Study participants were enrolled into the study from July 11, 2022 to August 22, 2023 at the Vaccine Evaluation Clinic in the National Institutes of Health Clinical Center. Data were collected through September 17, 2024.
Outcomes	The primary objective of the trial was to evaluate the safety and tolerability of a 2-dose vaccination regimen of mRNA-1215 at doses of 10 mcg, 25 mcg, 50 mcg or 100 mcg administered IM, given at a 4-week interval. For safety monitoring, all study participants were observed for a minimum of 30 minutes following each vaccination. Vital signs (temperature, blood pressure, pulse and respiratory rate) and assessment of local reactogenicity were performed after each product administration. Participants reported solicited local and systemic reactogenicity for the first 7 days following each vaccination. Adverse events (AEs) were collected for the first 28 days after each vaccination, while serious adverse events (SAEs) and new chronic medical conditions were recorded throughout the trial. The secondary objective of the trial was to evaluate antibody responses to the mRNA-1215 vaccine at doses of 10 mcg, 25 mcg, 50 mcg or 100 mcg at 2 weeks after last product administration. Serum samples were collected at protocol-specified timepoints for immunogenicity analysis of vaccine-induced antibody responses by Nipah virus IgG enzyme-linked immunosorbent assay (ELISA).

Plants

Seed stocks	*Report on the source of all seed stocks or other plant material used. If applicable, state the seed stock centre and catalogue number. If plant specimens were collected from the field, describe the collection location, date and sampling procedures.*
Novel plant genotypes	*Describe the methods by which all novel plant genotypes were produced. This includes those generated by transgenic approaches, gene editing, chemical/radiation-based mutagenesis and hybridization. For transgenic lines, describe the transformation method, the number of independent lines analyzed and the generation upon which experiments were performed. For gene-edited lines, describe the editor used, the endogenous sequence targeted for editing, the targeting guide RNA sequence (if applicable) and how the editor was applied.*
Authentication	*Describe any authentication procedures for each seed stock used or novel genotype generated. Describe any experiments used to assess the effect of a mutation and, where applicable, how potential secondary effects (e.g. second site T-DNA insertions, mosiacism, off-target gene editing) were examined.*

Flow Cytometry

Plots

Confirm that:

- [x] The axis labels state the marker and fluorochrome used (e.g. CD4-FITC).
- [x] The axis scales are clearly visible. Include numbers along axes only for bottom left plot of group (a 'group' is an analysis of identical markers).
- [x] All plots are contour plots with outliers or pseudocolor plots.
- [x] A numerical value for number of cells or percentage (with statistics) is provided.

Methodology

Sample preparation	B cells: Cryopreserved PBMC were thawed, washed briefly with phenol-free RPMI/4% heat inactivated newborn calf serum (R&D Systems) and incubated with aqua live/dead fixable dead cell stain kit (Thermo Fisher Scientific) for 20 minutes at RT.

Cells were stained with the following antibodies (monoclonal unless indicated) for 20 minutes at RT: IgD FITC [1:160] (goat polyclonal, Southern Biotech), IgM PerCP-Cy5.5 [1:20] (clone G20-127, BD Biosciences), IgA Dylight 405 [1:80] (goat polyclonal, Jackson Immunoresearch Inc), CD20 BV570 [1:40] (clone 2H7, Biolegend), CD27 BV650 [1:20] (clone O323, Biolegend), CD14 BV785 [1:80] (clone M5E2, Biolegend), CD16 BUV496 [1:80] (clone 3G8, BD Biosciences), CD4 BUV737 [1:320] (clone SK3, BD Biosciences), CD19 APC [1:80] (clone J3-119, Beckman), IgG Alexa 700 [1:20] (clone G18-145, BD Biosciences), CD3 APC-Cy7 [1:40] (clone SP34-2, BD Biosciences), CD38 PE [1:160] (clone OKT10, Caprico Biotechnologies), CD21 PE-Cy5 [1:40] (clone B-ly4, BD Biosciences) and CXCR5 PE-Cy7 [1:40] (clone MU5UBEE, Thermo Fisher Scientific). Stained cells were then incubated with matched Pre-F trimer and G trimer probe pairs: streptavidin-BUV661 (BD Biosciences) labeled Bangladesh Pre-F probe [1:25] and streptavidin-BUV395 labeled Malaysia Pre-F probe [1:25], and streptavidin-BUV661 (BD Biosciences) labeled Bangladesh G probe [1:25] and streptavidin-BUV395 labeled Malaysia G probe [1:25] for 30 minutes at 4°C (protected from light). The Pre-F probe used in these assays (GenBank AJ627196) is derived from a NiV Malaysia 1999 isolate that differs by only one amino acid, at position T348M, from AJ564621, the isolate sequence used for mRNA-1215. The NiV Malaysian G probe matched the sequence of G in the vaccine. For the Bangladesh probes, both the Pre-F and G amino acid sequences were derived from the 2004 sequence Genebank MK673564. Cells were washed and fixed in 0.5% formaldehyde (Tousimis Research Corp) prior to data acquisition.

T cells: Cryopreserved PBMCs were thawed and incubated overnight in at 37°C/5% CO2. After incubation, cells were stimulated with Malaysia Pre-F or Malaysia G peptide pools [Malaysia 1999 isolate NV/MY/99/VRI-2794 (GenBank AJ564621); JPT Peptides] at a final concentration of 2 µg/ml in the presence of 3 mM monensin for 6 hours. The Pre-F and G peptide pools are comprised of 123 and 108 individual peptides, respectively, as 15mers overlapping by 11 amino acids in 100% DMSO. Negative controls received an equal concentration of DMSO to that of peptide pools (final concentration of 0.5%). Cells were stained with the following monoclonal antibodies: CD3 APC-Cy7 [1:160] (clone SP34.2, BD Biosciences), CD4 PE-Cy5.5 [1:80] (clone SK3, Thermo Fisher), CD8 BV570 [1:40] (clone RPA-T8, BioLegend), CD45RA PE-Cy5 [1:2500] (clone 5H9, BD Biosciences), CCR7 BV650 [1:10] (clone G043H7, BioLegend), CXCR5 PE [1:10] (clone MU5UBEE, Thermo Fisher), CXCR3 BV711 [1:20] (clone 1C6/CXCR3, BD Biosciences), PD-1 BUV737 [1:20] (clone EH12.1, BD Biosciences), ICOS Pe-Cy7 [1:80] (clone C398.4A, BioLegend), CD69 ECD [1:40] (cloneTP1.55.3, Beckman Coulter), IFN-g Ax700 [1:320] (clone B27, BioLegend), IL-2 BV750 [1:40] (clone MQ1-17H12, BD Biosciences), IL-4 BB700 [1:20] (clone MP4-25D2, BD Biosciences), TNF-FITC [1:80] (clone Mab11, BD Biosciences), IL-13 BV421 [1:20] (clone JES10-5A2, BD Biosciences), IL-17 BV605 [1:20] (clone BL168, BioLegend), IL-21 Ax647 [1:10] (clone 3A3-N2.1, BD Biosciences), and CD154 BV785 [1:20] (clone 24-31, BioLegend). Aqua live/dead fixable dead cell stain kit (Thermo Fisher Scientific) was used to exclude dead cells.

Instrument

Samples were acquired on an BD FACSymphony flow cytometer.

Software

Samples were analyzed using FlowJo version 10.10.0 (Treestar, Inc., Ashland, OR).

Cell population abundance

N/A

Gating strategy

B cell gating strategy is shown in Extended Data Figure 2; T cell gating strategy is shown in Extended Data Figure 3.

☒ Tick this box to confirm that a figure exemplifying the gating strategy is provided in the Supplementary Information.
